# Synthesis and Evaluation of Spumigin Analogues Library with Thrombin Inhibitory Activity

**DOI:** 10.3390/md16110413

**Published:** 2018-10-27

**Authors:** Aleš Žula, Izabela Będziak, Danijel Kikelj, Janez Ilaš

**Affiliations:** 1 Faculty of Pharmacy, University of Ljubljana, Aškerčeva 7, 1000 Ljubljana, Slovenia; ales.zula@gmail.com (A.Ž.); izabedziak@gmail.com (I.B.); danijel.kikelj@ffa.uni-lj.si (D.K.); 2 Faculty of Pharmacy, The Poznan University of Medical Sciences, ul. Fredry 10, 61-701 Poznań, Poland

**Keywords:** marine products, natural peptides, peptidomimetics, thrombin inhibition

## Abstract

Spumigins are marine natural products derived from cyanobacteria *Nodularia spumigena*, which mimics the structure of the d-Phe-Pro-Arg sequence and is crucial for binding to the active site of serine proteases thrombin and factor Xa. Biological evaluation of spumigins showed that spumigins with a (2*S*,4*S*)-4-methylproline central core represent potential lead compounds for the development of a new structural type of direct thrombin inhibitors. Herein, we represent synthesis and thrombin inhibitory activity of a focused library of spumigins analogues with indoline ring or l-proline as a central core. Novel compounds show additional insight into the structure and biological effects of spumigins. The most active analogue was found to be a derivative containing l-proline central core with low micromolar thrombin inhibitory activity.

## 1. Introduction

**Figure 1:**
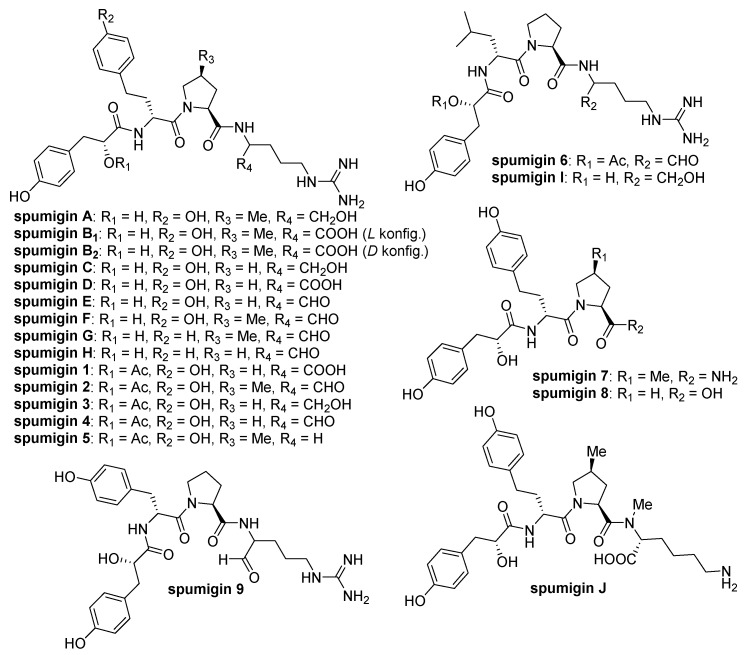
Structures of isolated spumigins.

**Figure 2:**
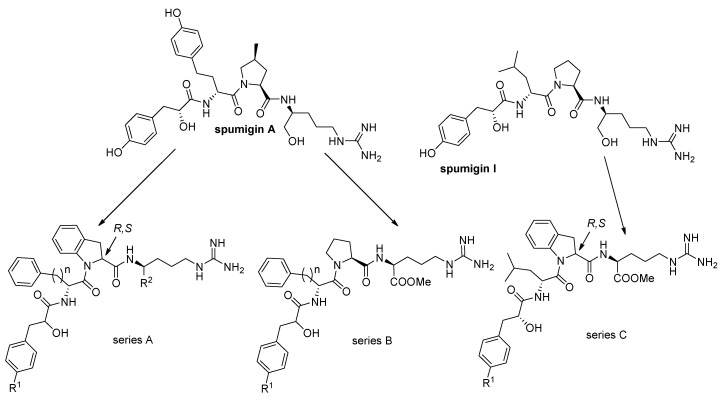
Different types of designed spumigin A analogues.

Cyanobacteria’s secondary metabolites are mostly peptides or possess a peptidic substructure and are products of a non-ribosomal biosynthetic pathway [1], with potential use in anti-cancer and anti-bacterial therapy. Cyanobacteria *Nodularia spumigena* produces a large number of different secondary metabolites among which the most known and investigated are spumigins, aeruginosins and nodularins [2,3]. Nodularins play an important role in the reproduction and survival of the *N. spumigena*, while for the spumigins it has not yet been fully known what their functions are. Spumigins were first isolated in 1997 and till year 2009, nine different spumigins had been described and structurally characterized [4]. After 2009 isolation, structural characterization and biological evaluation of spumigins became a more interesting field, resulting in 11 new spumigins. Spumigins are linear tetrapeptides composed of four subunits: N-terminal hydroxyl group containing lactic acid derivative, hydrophobic amino acid, proline core and the C-terminal guanidino group. d-hydroxyphenyllactic acid or d-hydroxyphenylacetyllactic acid are present at the N-terminal part, followed by hydrophobic amino acids such as d-homotyrosine, d-homophenylalanine, d-tyrosine or d-leucine. l-proline or (2*S*,4*S*)-4-methylproline are present as the central core and the C-terminal part consists of arginine, argininal, argininol or methyllysine (Figure 1) [5,6]. Spumigins with (2*S*,4*S*)-4-methylproline central core possess inhibitory activity to serine proteases, especially thrombin and trypsin in a low micromolar range (Table 1) [7]. Moreover, spumigins in their structures mimic the d-Phe-Pro-Arg sequence which is crucial for binding to the active site of thrombin (Figure 2) [8]. Docking of the spumigin J to thrombin demonstrated that the amino group of methyllysine binds to the S1 pocket of the thrombin active site, while the (2*S*,4*S*)-4-methylproline ring was bound in the hydrophobic pocket S2 and homotyrosine established the hydrophobic interactions with a hydrophobic aryl-S3 binding site [5]. Therefore, spumigins with (2*S*,4*S*)-4-methylproline central core represent interesting starting compounds for the development of a new structural type of direct thrombin inhibitors [9,10,11,12]. Herein, we report a series of new types of direct thrombin inhibitors based on the tetrapeptide structure of spumigins, where the (2*S*,4*S*)-4-methylproline central core was replaced in first series with an indoline ring as a more hydrophobic and rigid core, and in the second series of analogues with a more flexible l-proline core.

So far, only three spumigins were reported possessing thrombin inhibition activity with activity in a low micromolar range. Therefore, structure activity relationship (SAR) of spumigins as thrombin inhibitors is incomplete. A library of spumigins analogues was prepared where (2*S*,4*S*)-4-methylproline as a central core was initially replaced with more hydrophobic scaffold, indoline ring, and modifications were performed at the d-homophenylalanine, *p*-hydroxy-d-phenyllactic acid and at the arginine side of the spumigins structure. Indoline ring as a central scaffold increases the rigidity of analogues and may influence the orientation of pharmacophoric groups. In an attempt to better understand the SAR of spumigins-based analogues, analogues with flexible l-proline (series B), or with a rigid indole ring as a central scaffold (series A), thereby reducing the number of stereogenic centres, were also prepared (Figure 2). To investigate the importance of the P3 scaffold, Spumigin I analogues containing d-leucine were also prepared (series C).

## 2. Chemistry

**Scheme 1:**
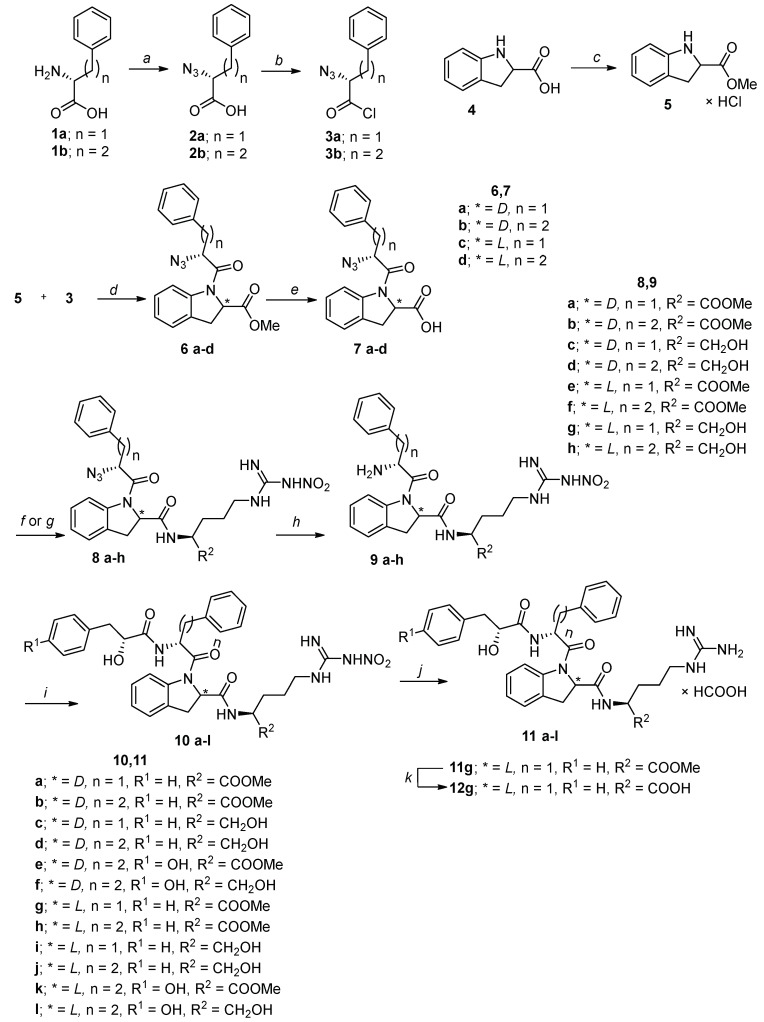
Reagents and conditions of the synthesis of series A compounds: (*a*) 1*H*-imidazole-1-sulfonyl azide hydrochloride, K_2_CO_3_, CuSO_4_·5H_2_O, rt, 17 h; (*b*) ClCOCOCl, DCM, DMF, reflux, 2 h; (*c*) SOCl_2_, MeOH, reflux, 12 h; (*d*) CsCO_3_, CH_3_CN, 0 °C, 45 min; (*e*) LiOH, THF, rt, 2 h; (*f*) coupling reaction type A: AA derivative, Et_3_N, EDC, HOBt, DMF, rt, 12h; (*g*) coupling reaction type B: AA derivative, NMM, TBTU, DMF, rt, 13 h; (*h*) Pd/C, H_2_, EtOH, 8 h; (*i*) coupling reaction type C: d-Pla or (±)-Hpla, NMM, TBTU, DMF, rt, 16 h; (*j*) Pd(OH)_2_/C, H_2_, EtOH, HCOOH, 35 °C, 12 h; (*k*) pig liver esterase (148 U/mg protein), buffer (pH 7)/DMSO (10:1), 24 h, rt; * Absolute configuration of compounds was not experimentally determined; assigned configuration on indoline chiral centre is based on calculations of biological activity and measured optical rotation. rt—room temperature.

Synthesis of series A spumigins analogues (Scheme 1) started from methyl 1*H*-indoline-2-carboxylate, where in the first step, the *N*-acylation of methyl 1*H*-indoline-2-carboxylate hydrochloride (**5**) was achieved with protected amino acid chloride **3** in the presence of an inorganic base. Other acylation procedures such as coupling with TBTU, EDC, BOP and acylation with amino acid anhydride did not lead to the desired compounds **6**. The key steps in the synthesis of series A compounds was in the selection of a proper amino acid protecting group. The substitution of the amino group of compound **1** with 1*H*-imidazole-1-sulfonyl azide hydrochloride [13] gave azido protected amino acids **2** in good yield (70–72%). Compounds **2** were then converted to acyl chloride **3** with oxalyl chloride. The acylation of racemic methyl 1*H*-indoline-2-carboxylate **5** with acyl chlorides **3** gave diasteromeric mixtures of compounds **6** in a 1:1 ratio of diastereomers, which were separated with normal phase flash chromatography to obtain pure diastereomers **6a**–**d** with 40% yield for each compound. The formed amide bond at position 1 of the indoline ring was labile under mild basic or acidic aqueous solutions or reduction with NaBH_4_ or LiAlH_4_, which affected the synthetic plan towards a series of spumigins analogues. To avoid the formation of the diketopiperazine [14] from prepared compounds **6** after reduction of the azido group, esters were cleaved to afford carboxylic acids **7** which were coupled with protected l-arginine or l-argininol to obtain compounds **8**, and at this stage the azido group of **8** was in the next step converted to the free amino group through Staudinger reaction or catalytic hydrogenation with Pd/C to afford unstable compounds **9** in the 90–95% yield. The catalytic hydrogenation was better in terms of yield (>90%), faster reaction and simpler isolation in comparison with the Staudinger reaction. Compounds **9** were used further without purification in the coupling reaction with unprotected d-phenyllactic acid and d,l-hydroxyphenyllactic acid in the presence of a TBTU coupling reagent and NMM base to afford compounds **10** in 50–62% yield. Other commercially available coupling reagents (EDC, HOBt and BOP) were also tested; however, in terms of yield and stereoselectivity, TBTU proved to be the best choice. Nitro protecting groups of l-arginine or l-argininol were cleaved under catalytic hydrogenation in the presence of Pd(OH)_2_/C and 10 equivalents of formic acid as catalysts at 35 °C to obtain compounds **11** in good yield (78–85%), while catalytic hydrogenation without formic acid or replacement of Pd(OH)_2_/C with Pd/C did not give the desired compounds **11**. Due to instability of the amide bond at position 1 on the indoline ring in mild basic or acidic aqueous solutions, the only appropriate procedure to prepare free carboxylic acid **12g** from **11g** was the use of pig liver esterase (PLE) (148 U/mg protein) in buffer solution pH 7 in good yield (80%) [15]. Despite the four stereogenic centres present, and 8 or 10 synthetic steps, the compounds **6**, **7**, **8**, **9**, **10a**–**d**, **10g**–**j**, **11a**–**d**, **11g**–**j** were prepared as pure diastereomers, which in the whole series of compounds showed a consistent pattern of chemical shifts in the ^1^H NMR spectra (indoline proton on position 2 had a constantly higher shift for diasteroisomer, which eluted first, e.g., 5.06 ppm for **6a** and 4.51 ppm for **6c**, 5.50 ppm for **7a** and 4.41 ppm for **7c**) and the same behaviour in the thin layer and column chromatography. Under normal phase chromatographic conditions (toluene, hexane and ethyl acetate = 6:3:0.5) diasteroisomer assigned as d-isomer was eluted first. Absolute configuration of the diastereomers on the indoline carbon C-2 was not determined. The assignment of configuration (l, d) on indoline carbon C-2 was done on the basis of the literature data for the characterization and biological evaluation of prepared compounds [2,4].

**Scheme 2:**
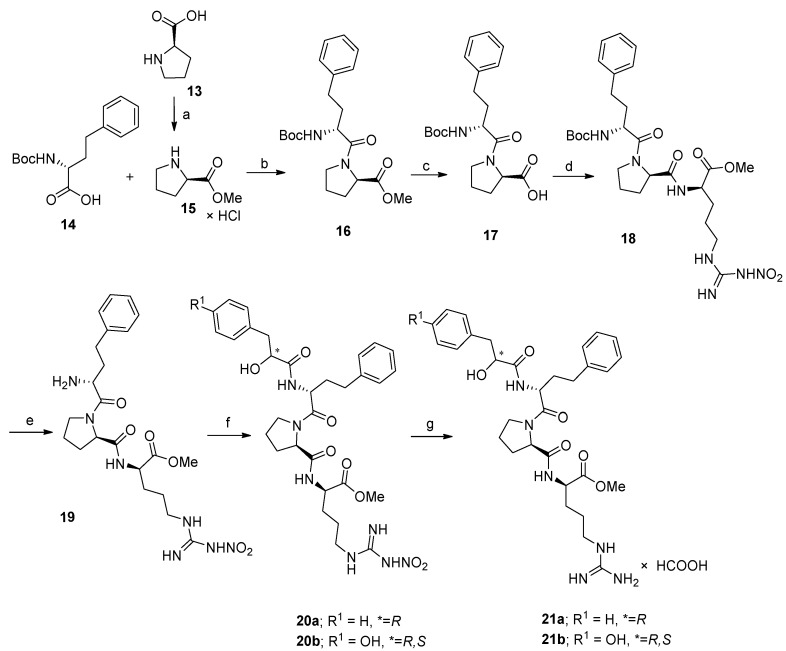
Reagents and conditions of the synthesis of series B compounds: (*a*) SOCl_2_, MeOH, reflux, 12 h; (*b*) EDC, HOBt, NMM,12 h, rt; (*c*) LiOH, THF, 2 h; (*d*) EDC, HOBt, NMM, l-Arg(NO_2_) methyl ester hydrochloride, 12 h; (*e*) HCl/MeOH, 8 h; (*f*) d-Pla or (±)-Hpla, TBTU, NMM, 6 h; (*g*) Pd(OH)_2_/C, H_2_, HCOOH, EtOH, 10 h.

The series B of spumigins analogues contains l-proline as a central core. Compounds of this series were prepared starting from l-proline methyl ester (**15**), which was coupled with Boc protected d-amino acids **14** in the presence of EDC and HOBt coupling reagents in 78–82% yield. In the next step, methyl esters **16** were cleaved with lithium hydroxide in methanol and the resulting carboxylic acids coupled with protected l-arginine used EDC/HOBt standard procedure to obtain compounds **18** in good yield (75–77% for both steps). The Boc protecting group of compounds **18** was cleaved under HCl/MeOH conditions in 95% yield to obtain compounds **19**. d-phenyllactic or d,l-hydroxyphenyllactic acid was coupled to prepared compounds **19** in the presence of TBTU and NMM to give compounds **20**. In the last two steps, nitro protecting groups were easily removed by hydrogenolysis and esters by mild basic conditions with lithium hydroxide to obtain the desired compounds **21a**–**b**. (Scheme 2).

**Scheme 3:**
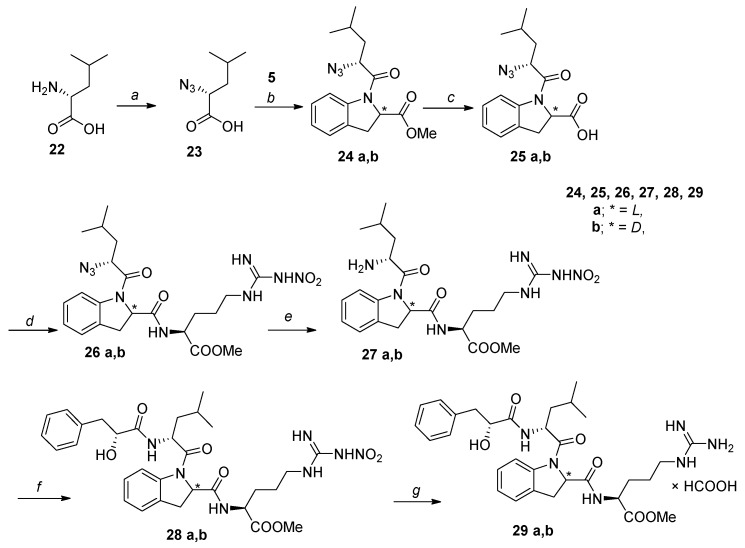
Reagents and conditions of the synthesis of series A compounds: (*a*) 1*H*-imidazole-1-sulfonyl azide hydrochloride, K_2_CO_3_, CuSO_4_·5H_2_O, rt, 17 h; (*b*) (*i*) ClCOCOCl, DCM, DMF, reflux, 2 h; (*ii*) CsCO_3_, CH_3_CN, 0 °C, 45 min; (c) LiOH, THF, rt, 1 h; (*d*) l-Arg(NO_2_)-OMe, Et_3_N, EDC, HOBt, DMF, rt, 12 h; (*e*) Pd/C, H_2_, EtOH, 7 h; (*f*) d-Pla, NMM, TBTU, DMF, rt, 16 h; (*j*) Pd(OH)_2_/C, H_2_, EtOH, HCOOH, 35 °C, 12 h; * Absolute configuration of the compounds was not experimentally determined, assigned configuration on indoline chiral centre is based on calculations of biological activity and measured optical rotation.

The series C was prepared (Scheme 3) following a similar strategy to series A. The substitution of the amino group of d-leucine with 1*H*-imidazole-1-sulfonyl azide hydrochloride gave azido-protected amino acids, which was converted to acyl chloride with oxalyl chloride. The acylation of racemic methyl 1*H*-indoline-2-carboxylate **5** with acyl chlorides gave diastereomeric mixtures of compounds **24** in a 1:1 ratio of diastereomers, which were separated with normal phase flash chromatography to obtain pure diastereomers **24a**–**b** with 40% yield for each compound. Esters were cleaved to afford carboxylic acids **25a**–**b** which were coupled with nitro protected l-arginine to obtain compounds **26a**–**b**, and the azido group was converted to the free amino group through catalytic hydrogenation with Pd/C to afford unstable compounds **27a**–**b**, which were immediately coupled with unprotected d,l-hydroxyphenyllactic acid in the presence of TBTU coupling reagent and NMM base to afford compounds **28a**–**b** in 83% yield. Nitro-protecting groups of l-arginine were cleaved under catalytic hydrogenation in the presence of Pd(OH)_2_/C and 10 equivalents of formic acid as catalyst at 35 °C to obtain compounds **29-a**,**b** in moderate yield (46%). As in series A, the compounds were prepared as pure diastereomers, and the assignment of configuration (l, d) on indoline carbon C-2 was done based on the literature data for the characterization and biological evaluation of prepared compounds.

## 3. Biological Evaluation

**Figure 3:**
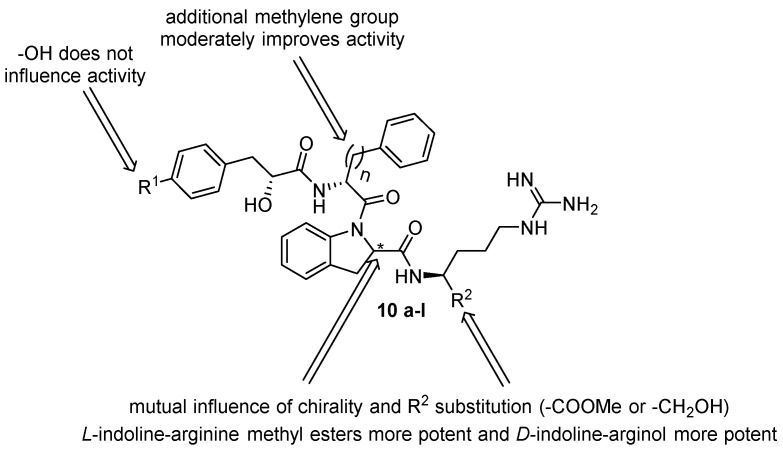
Summary of SAR results.

The library of 17 spumigins analogues was biologically evaluated for the inhibition of serine proteases thrombin, factor Xa and trypsin (Table 2 and Figure 3). The obtained results indicate that l-arginine esters derivatives possessing l-isomer indoline ring (**11g**,**h**,**k**) are more potent or similar in activities to d-isomers (**11a**,**b**,**e**) in thrombin inhibition, while the trend is opposite with l-argininol derivatives, where compounds possessing d-isomer indoline ring (**11c**,**d**,**f**) are more potent or similar in activities than l-isomers (**11i**,**j**,**l**) in thrombin inhibition. Indoline isomerism has an even greater impact on trypsin inhibitory activity, where compounds possessing the d-isomer indoline ring are more potent than l-isomers on trypsin inhibitory activity; only l-arginol derivatives **11c** and **11i** have comparable activity. There is not a significant difference between compounds possessing the d-isomer indoline ring compered to compounds possessing the l-isomer indoline ring on factor Xa inhibitory activity. The 4-Hydroxy group on d-phenyllactic acid does not contribute to more potent thrombin or trypsin inhibitory activity (d-Pla derivative **11h** with thrombin K_i_ = 24 ± 8 μM versus (*±*)-Hpla derivative **11k** with thrombin K_i_ = 34 ± 10 μM). Homophenylalanine, with one additional methylene group as compared with phenylalanine in the indoline series, in some cases, contributes to better thrombin inhibition; homophenylalanine derivative **11d** with thrombin K_i_ = 11 ± 4 μM is eight times more potent than phenylalanine derivative **11c** with thrombin K_i_ = 92 ± 20 μM and homophenylalanine derivative **11h** with thrombin K_i_ = 24 ± 8 μM is twice as potent than phenylalanine derivative **11g** with thrombin K_i_ = 50 ± 9 μM. The additional methylene group does not affect factor Xa inhibitory activity, while it contributes to trypsin inhibitory activities with twofold improvement in the whole series, except for phenylalanine derivative **11j** being more potent than the homophenylalanine derivative **11j**. Alcohol derivatives containing the l-argininol moiety are in general from one to four times more potent than l-arginine esters (l-argininol derivative **11d** with thrombin K_i_ = 11 ± 4 μM versus l-arginine ester **11b** with thrombin K_i_ = 44 ± 7 μM), with the exception of carboxylic ester **11g** being more potent than the l-argininol derivative **11i**. Free carboxylic acid on the l-arginine moiety decreases thrombin inhibitory activity by a factor of two (**12g**, thrombin K_i_ = 98 ± 26 μM) in comparison with the corresponding methyl ester (**11g**, thrombin K_i_ = 44 ± 7 μM), while acid is a more potent trypsin inhibitor, being the best trypsin inhibitor (K_i_ = 0.28 ± 0.04 μM) within all compounds. Thrombin inhibition of spumigin A in terms of the suitability of indoline ring as a bioisosteric replacement of (2*S*,4*S*)-4-methylproline cannot be directly compared because compounds with homotyrosine, which is present in spumigin A, were not prepared. However, test results indicate that with indoline ring replacement of (2*S*,4*S*)-4-methylproline, no improvement in activity was achieved. Replacement of (2*S*,4*S*)-4-methylproline with l-proline has proved to be more productive as a replacement of (2*S*,4*S*)-4-methylproline with indoline, as proved by compounds **21a** and **21b**, resulting in a compound with potent thrombin inhibitory activity (K_i_ = 3.8 ± 0.4 μM and K_i_ = 3.9 ± 0.4 μM). Direct comparison shows that the indoline ring compared to proline results with sixfold/ninefold lower activity (**11h** vs. **21a** and **11k** vs. **21b**). This can be attributed to the fact that due to the presence of the aromatic ring of phenylalanine and homophenylalanine, the additional aromatic ring of indoline causes sterical clashes. In the case of indoline compounds, having d-phenylalanine (compound **11a**) substituted with d-leucine (spumigin I analogues) compounds possess ninefold better thrombin inhibitory activity (**29a**, thrombin K_i_ = 5.6 ± 0.3 μM).

## 4. Discussion

A focused library of spumigins analogues based on a mimicked d-Phe-Pro-Arg sequence was designed, synthesized and biologically evaluated for thrombin inhibitory activity. (2*S*,4*S*)-4-methylproline central core of spumigins was replaced with other more flexible or more rigid scaffolds, while the configuration of amino acids in spumigins structures were retained. The key step in the synthesis was proper protection of the amino group of phenyl or homophenylalanine amino acid. For the synthesis of series A analogues, the amino group of phenylalanine and homophenylalanine were simply converted to the azido group with reagent 1*H*-imidazole-1-sulfonyl azide hydrochloride and this protection was compatible with all subsequent reaction steps, enabling an efficient synthesis of diastereomerically pure spumigin A analogues **10**–**12**. The most potent thrombin inhibitors derived from this series were compounds **21a** and **21b** and it can be concluded that in the investigated series, l-proline is the most optimal central scaffold for thrombin inhibition. The generated structure activity relationship represents valuable information for future optimization towards a more potent thrombin inhibitor, based on the spumigin structure. Simple and facile synthetic strategy can be employed for additional preparation of synthetic analogues of spumigins.

## 5. Chemistry–General

The chemicals obtained from Acros (Geel, Belgium), Aldrich Chemical Co. (Saint Quentin Fallavier, France), and Fluka (Saint Louis, MO, USA) were used without further purification. The reactions were monitored with TLC on silica gel Merck 60 F254 plates (0.25 mm) and visualization was performed with UV light, ninhydrin and phosphomolybdic acid. The purification of compounds was carried out with column chromatography on silica gel 60 (particle size 240–400 mesh). HPLC analyses were performed on an Agilent Technologies 1100 instrument with a G1365B UV-VIS detector (Agilent, Santa Clara, CA, USA), a G1316A thermostat and a G1313A autosampler using a Phenomenex Luna 5 μm C18 column (4.6 × 150 mm or 4.6 × 250 mm) and flow rate of 1.0 mL/min. Low resolution ESI-mass spectra were obtained with an Expression CMS spectrometer (Advion, Ithaca, NY, USA). Melting points were determined on a Reichert hot stage microscope. ^1^H spectra were measured at 400 and 600 MHz and ^13^C NMR at 100 and 150 MHz on a Bruker AVANCE III 400 (Bruker Biospin, Falländen, Switzerland)and Varian NMR System 600 MHz spectrometer (Varian, Palo Alto, CA, USA) in DMSO-*d*_6_, MeOH-*d*_4_ and CDCl_3_ solution, with TMS as the internal standard. IR spectra were recorded on a Perkin-Elmer Spectrum BX FT-IR spectrometer (PerkinElmer, Inc., Hebron, KY, USA) or Thermo Nicolet Nexus 470 ESP FT-IR spectrometer (Thermo Nicolet, Madison, WI, USA). Mass spectra were determined on a VGAnalytical Autospec Q mass spectrometer (VG Analytical, Manchester, UK). The HPLC purity of tested compounds was determined to be >95% (254 nm). See Supplementary Materials.

## 6. Synthetic Procedures

### 6.1. General Procedure of Coupling Reaction, Type A

Carboxylic acid (1.8 mmol) and free amine (1.89 mmol) were dissolved in 15 mL dry DMF and the mixture was cooled down to 0 °C and then triethylamine, HOBt (2.16 mmol) and EDC (2.34 mmol) were added under an argon atmosphere. The resulting mixture was stirred at 0 °C for 2 h, allowed to come to room temperature, and the stirred at room temperature overnight. After the completion of the reaction (TLC), the solvent was evaporated, and the crude product was dissolved in 30 mL of dichloromethane. The organic phase was washed with saturated NaHCO_3_ (2 × 20 mL) and brine (1 × 20 mL), dried with Na_2_SO_4_ and evaporated under reduced pressure to obtain orange-brown residue, which was purified by flash column chromatography (SiO_2_) eluting with dichloromethane and methanol.

### 6.2. General Procedure of Coupling Reaction; Type B

Carboxylic acid (1.8 mmol), TBTU (2.16 mmol) and *N*-methylmorpholine (395 μL, 3.6 mmol) were dissolved in 15 mL of anhydrous dimethylformamide and stirred under an argon atmosphere at rt for 1 h. Prepared mixture was added dropwise to a stirred solution of free amine (1.89 mmol, 1.05 eq.) and *N*-methylmorpholine (395 μL, 3.6 mmol) in 15 mL of anhydrous dimethylformamide at 0 °C. After 1 h, the mixture was warmed to room temperature and stirred under argon for 12 h at the same temperature. The solvent was evaporated under reduced pressure and the crude product was dissolved in 30 mL of dichloromethane. The organic phase was washed with saturated NaHCO_3_ (2 × 20 mL) and brine (1 × 20 mL), dried with Na_2_SO_4_ and evaporated under reduced pressure. The residue was purified by flash column chromatography (SiO_2_) eluting with dichloromethane and methanol to obtain pure compounds.

### 6.3. General Procedure of Coupling Reaction; Type C

Free amine (1.2 mmol), carboxylic acid (1.26 mmol, 1.05 eq.) and NMM (2.64 mmol) were dissolved in 15 mL of dry DMF and cooled to 0 °C. TBTU (1.44 mmol) was added dropwise in 5 mL of DMF over a period of 2 h under an argon atmosphere. The resulting mixture was stirred for 4 h more at 0 °C and then was allowed to come to room temperature and stirred at room temperature for an additional 12 h. The solvent was evaporated, and the crude product was dissolved in 30 mL of dichloromethane. The organic phase was washed with saturated NaHCO_3_ (2 × 20 mL) and brine (1 × 20 mL), dried with Na_2_SO_4_ and evaporated under reduced pressure. Residue was purified by flash column chromatography (SiO_2_) eluting with dichloromethane and methanol to obtain pure compounds.

### 6.4. General Procedure of Synthesis of the d-2-Azido-3-phenylalkanoic acid from Corresponding Amino Acids 

d-amino acid (10 mmol) was dissolved in 60 mL of dry methanol. Under an argon atmosphere potassium carbonate (40 mmol) and CuSO_4_·5H_2_O (0.1 mmol) were added and the mixture was stirred for 15 min. 1*H*-imidazole-1-sulfonyl azide hydrochloride (12 mmol) was added over a period of 30 min and the resulting mixture was stirred for 17 h at ambient temperature and under an argon atmosphere. Methanol was removed under reduced pressure and 50 mL of water was added to the crude product. The pH was adjusted to 3 with 1 M HCl and extracted with ethyl acetate (3 × 40 mL). The organic phase was washed with brine (1 × 30 mL), dried with Na_2_SO_4_ and evaporated under reduced pressure, and the resulting oil residue was purified by flash column chromatography (SiO_2_) eluting with dichloromethane and methanol with 1% of acetic acid

### 6.5. General Procedure for Preparation of d-2-Azido-3-phenylalkanoyl Chloride

To a cooled solution (0 °C) of d-azido amino acid (6 mmol) in 80 mL of dry dichloromethane, four drops of dry DMF and dropwise oxalyl chloride (7.2 mmol) in 20 mL of dry dichloromethane were added. The resulting mixture was stirred for an additional 30 min at 0 °C and then under reflux for 2 h under an argon atmosphere. The solvent was evaporated under reduced pressure, washed with hexane and the resulting oil was used in the next reaction.

### 6.6. General Procedure for Acylation of Methyl Indoline-2-Carboxylate

To a cooled solution (0 °C) of methyl indoline-2-carboxylate (5 mmol) in 60 mL of dry acetonitrile, CsCO_3_ (11 mmol) and dropwise freshly prepared d-2-azido-3-phenylalkanoyl chloride (6 mmol) in 20 mL of dry acetonitrile were added under an argon atmosphere. After 30 min, the mixture was warmed to room temperature and stirred under argon for 1 h at the same temperature. The solvent was removed under reduced pressure and the resulting crude product was dissolved in 80 mL of dichloromethane. The organic phase was washed with brine (2 × 40 mL), dried with Na_2_SO_4_ and evaporated under reduced pressure to obtain a yellow crude product (mixture of diastereomers) which was purified and separated by flash column chromatography (SiO_2_), eluting with three component mobile phases consisting of toluene, hexane and ethyl acetate (6:3:0.5) to afford two completely separated diastereoisomers.

### 6.7. General Procedure of Ester Hydrolysis; Type A

Methyl esters were dissolved in 10 mL of THF and 1 M LiOH (5 mL, 5 mmol) was added at room temperature. After 1 h, THF was evaporated and 20 mL of water was added to the mixture. The pH was adjusted to three with 1 M HCl and extracted with ethyl acetate (3 × 20 mL). The organic phase was washed with brine (1 × 20 mL), dried with Na_2_SO_4_ and evaporated under reduced pressure to obtain pure carboxylic acid.

### 6.8. General Procedure of Ester Hydrolysis, Type B 

Esters (0.1 mmol) were dissolved in 3 mL of DMSO/phosphate buffer pH 7 mixture (ratio 1:10) and pig liver esterase (PLE) (10 μL of 9.2 mg/100 mL solution) were added at room temperature. After the completion of the reaction (TLC), the solvent was removed under reduced pressure and the crude product was purified by flash column chromatography (SiO_2_) eluting with ethyl acetate and methanol with 2% of formic acid to obtain the desired carboxylic acids.

### 6.9. General Procedure of Azido Groups Reduction

To a solution of azido compound (1.4 mmol) in 30 mL of anhydrous ethanol, palladium on activated charcoal (30 wt %) was added under an argon atmosphere. The mixture was flushed with H_2_ for 30 min, and sealed and stirred for 4 to 6 h under H_2_ atmosphere. After the completion of the reaction, the Pd/C was filtered, and ethanol was evaporated under reduced pressure to obtain a light brown residue which was used further without purification.

### 6.10. General Procedure of Nitro Group Cleavage on Protected l-Arginine or l-Argininol Derivatives

To the solution of the nitro group-protected compound (0.5 mmol) in 25 mL anhydrous ethanol, palladium hydroxide on activated charcoal (30 wt %) and formic acid (189 μL, 5 mmol) were added under an argon atmosphere. The prepared mixture was flushed with H_2_ for 30 min, sealed and stirred for 12 h under H_2_ atmosphere at 35 °C. After the completion of the reaction, the Pd(OH)_2_/C was filtered and ethanol was evaporated under reduced pressure. The resulting residue was purified by flash column chromatography (SiO_2_) eluting with ethyl acetate and methanol containing 2% of formic acid to obtain the desired deprotected compound.

*(R)-2-Azido-3-phenylpropanoic acid* (**2a**). Synthesized from **1a** (10 mmol) using the general procedure described in *6.4*. Brown oil, yield: 1.34 g (70%). ^1^H NMR (DMSO-*d*_6_, 400 MHz): δ 2.93 (dd, 1H, *J* = 14.1 Hz, *J* = 8.9 Hz, -CHCH_2_Ph), 3.12 (dd, 1H, *J* = 14.1 Hz, *J* = 5.0 Hz, -CHCH_2_Ph), 4.40 (dd, 1H, *J* = 8.9 Hz, *J* = 5.0 Hz, -CHCH_2_Ph), 7.25–7.34 (m, 5H, -CHCH_2_Ph), 13.45 (bs, 1H, -COOH) ppm. ^13^C NMR (DMSO-*d*_6_, 100 MHz): δ 171.24, 136.68, 129.18, 128.87, 128.32, 126.74, 125.29, 62.22, 36.63 ppm. IR(ATR) *ν*: 3031, 2106, 1714, 1497, 1455, 1418, 1260, 1221, 1081, 749, 697 cm^−1^.

*(R)-2-Azido-4-phenylbutanoic acid* (**2b**). Synthesized from **1b** (10 mmol) using the general procedure described in *6.4*. Brown oil, yield: 1.48 g (72%). ^1^H NMR (DMSO-*d*_6_, 400 MHz): δ 1.86–1.95 (m, 1H, -CHCH_2_CH_2_Ph), 2.00–2.09 (m, 1H, -CHCH_2_CH_2_Ph), 2.65–2.69 (m, 2H, -CHCH_2_CH_2_Ph), 4.08 (dd, 1H, *J* = 8.8 Hz, *J* = 4.6 Hz, -CHCH_2_CH_2_Ph), 7.19–7.23 (m, 3H, -CHCH_2_CH_2_Ph), 7.28–7.33 (m, 2H, -CHCH_2_CH_2_Ph), 13.40 (bs, 1H, -COOH) ppm. ^13^C NMR (DMSO-*d*_6_, 100 MHz): δ 172.19, 141.03, 128.92, 128.79, 126.57, 61.26, 33.03, 31.77 ppm. IR(ATR) *ν*: 3028, 2929, 2104, 1713, 1497, 1454, 1419, 1227, 1030, 910, 748, 698 cm^−1^.

*Methyl indoline-2-carboxylate hydrochloride* (**5**). To the cooled (0 °C) solution of 1*H*-indoline-2-carboxylic acid (1.63 g, 10 mmol) in 30 mL of methanol, SOCl_2_ (2.4 mL, 33.3 mmmol) dropwise in 10 mL of methanol was added. The resulting mixture was stirred at 0 °C for 1 h and then for 12 h under reflux. After completion of the reaction (TLC), the solvent was removed under reduced pressure and the crude product was washed with ether (3 × 40 mL) and hexane (3 × 40 mL) to obtain a pure compound **5** (2.01 g, 94%) as a brown solid. ^1^H NMR (DMSO-*d*_6_, 400 MHz): δ 3.13 (dd, 1H, *J* = 16.1 Hz, *J* = 6.3 Hz, -NHCHCH_2_-), 3.34 (dd, 1H, *J* = 16.1 Hz, *J* = 10.3 Hz, -NHCHCH_2_-), 3.69 (s, 3H, -COOMe), 4.51 (dd, 1H, *J* = 10.3 Hz, *J* = 6.3 Hz, -NHCHCH_2_-), 6.16 (bs, 2H, -NHCHCH_2_-), 6.70–6.73 (m, 2H, indoline Ar-H), 7.02 (dt, 1H, *J* = 8.0 Hz, *J* = 7.8 Hz, *J* = 1.1 Hz, indoline Ar-H), 7.09 (dd, 1H, *J* = 7.7 Hz, *J* = 1.0 Hz, indoline Ar-H) ppm. ^13^C NMR (DMSO-*d*_6_, 100 MHz): δ 171.88, 130.35, 128.23, 125.40, 123.64, 114.35, 59.44, 53.00, 49.04, 33.26 ppm. IR(ATR) *ν*: 3319, 2898, 2664, 2547, 2492, 1735, 1623, 1486, 1464, 1440, 1408, 1339, 1301, 1264, 1110, 1025, 1043, 916, 769, 680 cm^−1^.

*Methyl (R)-1-((R)-2-azido-3-phenylpropanoyl)indoline-2-carboxylate* (**6a**). Synthesized from **3a** (1.26 g, 6 mmol) and **5** (1.07 g, 5 mmol) using the general procedure described in *6.6*. Colourless oil, yield: 0.7 g (40%). [α]${}_{D}^{25}$ −1.18 (c 0.206, MeOH), ^1^H NMR (DMSO-*d*_6_, 400 MHz): δ 3.06–3.11 (m, 1H, -CHCH_2_Ph), 3.20–3.26 (m, 3H, -NHCHCH_2_- + -CHCH_2_Ph), 3.67 (s, 3H, -COOMe), 4.31–4.35 (m, 1H, -CHCH_2_Ph), 5.06–5.09 (m, 1H, -NHCHCH_2_-), 7.06–7.10 (m, 1H, indoline Ar-H), 7.22–7.35 (m, 7H, indoline Ar-H + -CHCH_2_Ph), 8.12 (d, 1H, *J* = 7.7 Hz, indoline Ar-H) ppm. ^13^C NMR (DMSO-*d*_6_, 100 MHz): δ 173.24, 169.66, 143.19, 138.13, 131.29, 130.42, 130.28, 129.71, 128.67, 128.08, 126.15, 125.75, 118.66, 63.19, 61.93, 53.66, 37.41, 33.97 ppm. HRMS for C_19_H_19_N_4_O_3_: calculated 351.1457; found 351.1459. IR(ATR) *ν*: 3330, 3029, 2953, 2104, 1736, 1662, 1599, 1481, 1464, 1406, 1308, 1230, 1205, 1667, 1080, 1022, 1006, 931, 833, 749, 698, 666 cm^−1^.

*(R)-1-((R)-2-Azido-3-phenylpropanoyl)indoline-2-carboxylic acid* (**7a**). Synthesized from **6a** (665 mg, 1.9 mmol) using the general procedure described in *6.7*. White solid, yield: 606 mg (95%). ^1^H NMR (DMSO-*d*_6_, 400 MHz): δ 3.10 (dd, 1H, *J* = 14.3, *J* = 10.2 Hz, -CHCH_2_Ph), 3.20 (dd, 1H, *J* = 14.4 Hz, *J* = 3.4 Hz, -CHCH_2_Ph), 3.29–3.41 (m, 1H, -NHCHCH_2_), 3.63 (dd, 1H, *J* = 16.7 Hz, *J* = 11.1 Hz, -NHCHCH_2_-), 4.22 (dd, 1H, *J* = 10.5 Hz, *J* = 3.4 Hz, -CHCH_2_Ph), 5.50 (dd, 1H, *J* = 10.5 Hz, *J* = 1.9 Hz, -NHCHCH_2_-), 7.11 (t, 1H, *J* = 7.3 Hz, indoline Ar-H), 7.23–7.38 (m, 7H, indoline Ar-H + -CHCH_2_Ph), 8.13 (d, 1H, *J* = 8.0 Hz, indoline Ar-H) ppm. HRMS for C_18_H_15_N_4_O_3_: calculated 335.1144; found 335.1142. IR(ATR) *ν*: 3029, 2926, 2104, 1736, 1709, 1661, 1627, 1599, 1480, 1463, 1409, 1341, 1260, 1188, 1168, 1078, 883, 747, 701, 639 cm^−1^.

*Methyl N^2^-((R)-1-((R)-2-azido-3-phenylpropanoyl)indoline-2-carbonyl)-N^ω^-nitro-l-argininate* (**8a**). Synthesized from **7a** (269 mg, 0.8 mmol) using the general coupling procedure type A. White solid, yield: 361 mg (82%). ^1^H NMR (DMSO-*d*_6_, 400 MHz): δ 1.56–1.84 (m, 4H, -CHCH_2_CH_2_CH_2_NH-), 3.05–3.07 (m, 2H, -CHCH_2_Ph), 3.13–3.17 (m, 3H, -CHCH_2_CH_2_CH_2_NH- + -NHCHCH_2_-), 3.61 (s, 3H, -COOMe), 3.72 (dd, 1H, *J* = 16.8 Hz, *J* = 11.0 Hz, -NHCHCH_2_-), 3.88 (dd, 1H, *J* = 8.8 Hz, *J* = 5.4 Hz, -CHCH_2_CH_2_CH_2_NH-), 4.21–4.26 (m, 1H, -CHCH_2_Ph), 5.34 (dd, 1H, *J* = 11.0 Hz, *J* = 2.7 Hz, -NHCHCH_2_-), 7.09 (t, 1H, *J* = 7.1 Hz, indoline Ar-H), 7.20–7.37 (m, 7H, indoline Ar-H + -CHCH_2_Ph), 8.14 (d, 1H, *J* = 7.9 Hz, indoline Ar-H), 8.57 (bs, 1H, guanidine-NH), 8.88 (d, 1H, *J* = 7.2 Hz, -CONH-) ppm. ^13^C NMR (MeOH-*d*_4_, 100 MHz): δ 172.39, 172.04, 168.20, 159.57, 142.48, 136.82, 129.82, 128.81, 128.74, 128.35, 128.18, 127.20, 126.74, 124.59, 124.27, 117.14, 62.00, 60.85, 52.54, 51.52, 40.22, 35.82, 34.03 ppm. HRMS for C_25_H_28_N_9_O_6_: calculated 550.2163; found 550.2160. IR(ATR) *ν*: 3286, 2928, 2104, 1739, 1626, 1597, 1535, 1482, 1408, 1256, 1211, 1151, 1019, 752, 669, 659 cm^−1^.

*Methyl N^2^-((R)-1-(((R)-2-hydroxy-3-phenylpropanoyl)-d-phenylalanyl)indoline-2-carbonyl)-N^ω^-nitro-l-argininate* (**10a**). Synthesized from **9a** (120 mg, 0.23 mmol) using the general coupling procedure type C. White solid, yield: 95 mg (62%). ^1^H NMR (DMSO-*d*_6_, 400 MHz): δ 1.58–1.83 (m, 4H, -CHCH_2_CH_2_CH_2_NH-), 2.77 (dd, 1H, *J* = 13.4, *J* = 3.6 Hz, -CH(OH)CH_2_Ph), 2.90 (d, 1H, *J* = 13.4 Hz, -CH(OH)CH_2_Ph), 3.00 (dd, 1H, *J* = 13.4 Hz, *J* = 11.1 Hz, -CHCH_2_Ph), 3.18–3.21 (m, 3H, -CHCH_2_CH_2_CH_2_NH- + CHCH_2_Ph), 3.62–3.69 (m, 4H, -COOMe + -NHCHCH_2_-), 4.00–4.06 (m, 1H, -NHCHCH_2_-), 4.25 (dd, 1H, *J* = 12.4 Hz, *J* = 7.2 Hz, -CHCH_2_CH_2_CH_2_NH-), 4.44 (t, 1H, *J* = 7.4 Hz, -CHCH_2_Ph), 5.45 (d, 1H, *J* = 6.1 Hz, -NHCHCH_2_-), 5.64 (dd, 1H, *J* = 11.1 Hz, *J* = 2.9 Hz, -CH(OH)CH_2_Ph), 7.06–7.29 (m, 13H, indoline Ar-H + -CHCH_2_Ph + -CH(OH)CH_2_Ph), 8.14 (d, 1H, *J* = 8.0 Hz, indoline Ar-H), 8.21 (d, 1H, *J* = 7.6 Hz, -CONH-), 8.58 (bs, 1H, guanidine-NH), 8.97 (d, 1H, *J* = 6.7 Hz, -CONH-) ppm. ^13^C NMR (MeOH-*d*_4_, 100 MHz): δ 173.51, 172.20, 171.83, 170,45, 142.94, 138.07, 137.75, 130.00, 129.24, 128.96, 128.13, 127.79, 127.15, 126.34, 125.92, 124.63, 123.82, 116.47, 71.91, 60.24, 55.99, 54.90, 52.61, 52.34, 51.99, 40.33, 36.18, 33.91, 27.69, 18.54 ppm. HRMS for C_34_H_38_N_7_O_8_: calculated 672.2782; found 672.2793. IR(ATR): 3296, 3028, 2494, 1736, 1631, 1625, 1529, 1483, 1404, 1263, 1150, 1088, 1026, 736, 698, 632 cm^−1^.

*Methyl ((R)-1-(((R)-2-hydroxy-3-phenylpropanoyl)-d-phenylalanyl)indoline-2-carbonyl)-l-argininate* (**11a**). Synthesized from **10a** (135 mg, 0.2 mmol) using the general procedure of nitro group cleavage described in *6.10*. White solid, yield: 109 mg (81%). [α]${}_{D}^{25}$ −0.45 (c 0.130, MeOH), ^1^H NMR (DMSO-*d*_6_, 600 MHz): δ 1.57–1.61 (m, 2H, -CHCH_2_CH_2_CH_2_NH-), 1.70–1.73 (m, 1H, -CHCH_2_CH_2_CH_2_NH-), 1.79–1.82 (m, 1H, -CHCH_2_CH_2_CH_2_NH-), 2.52 (dd, 1H, *J* = 8.4 Hz, *J* = 5.3 Hz, -CH(OH)CH_2_Ph), 2.77 (dd, 1H, *J* = 13.8 Hz, *J* = 3.9 Hz,-CH(OH)CH_2_Ph), 2.91 (dd, 1H, *J* = 13.7 Hz, *J* = 1.9 Hz, -CHCH_2_Ph), 2.99 (dd, 1H, *J* = 14.0 Hz, *J* = 10.8 Hz, -CHCH_2_Ph), 3.04–3.11 (m, 2H, -CHCH_2_CH_2_CH_2_NH-), 3.22 (dd, 1H, *J* = 16.9 Hz, *J* = 2.1 Hz, -NHCHCH_2_-), 3.60 (s, 3H, -COOMe), 3.65 (dd, 1H, *J* = 17.0 Hz, *J* = 11.2 Hz, -NHCHCH_2_-), 4.01 (dd, 1H, *J* = 8.4 Hz, *J* = 4.0 Hz, -CHCH_2_CH_2_CH_2_NH-), 4.18–4.21 (m, 1H, -CHCH_2_Ph), 4.42–4.46 (m, 1H, -NHCHCH_2_-), 5.65 (dd, 1H, *J* = 11.4 Hz, *J* = 3.0 Hz, -CH(OH)CH_2_Ph), 7.03–7.29 (m, 13H, -CH(OH)CH_2_Ph + -CHCH_2_Ph + indoline Ar-H), 8.13 (d, 1H, *J* = 8.1 Hz, indoline Ar-H), 8.20 (d, 1H, *J* = 8.0 Hz, -CONH-), 8.45 (s, 1H, HCOO^−^), 9.16 (t, 1H, *J* = 4.4 Hz, guanidine-NH), 9.31 (d, 1H, *J* = 6.7 Hz, -CONH-), ppm. ^13^C NMR (DMSO-*d*_6_, 150 MHz): δ 173.45, 172.09, 171.76, 170.40, 166.34, 157.35, 142.88, 138.07, 137.69, 129.93, 129.17, 128.85, 128.05, 127.70, 126.29, 125.81, 123.70, 116.38, 71.81, 60.17, 52.62, 52.29, 51.85, 40.24, 40.07, 36.10, 33.82, 27.50, 25.03 ppm. HRMS for C_34_H_41_N_6_O_6_: calculated 629.3088; found 629.3083. IR(ATR) *ν*: 3323, 3212, 2960, 1738, 1640, 1531, 1483, 1454, 1419, 1201, 1134, 1002, 837, 800, 752, 699, 628 cm^−1^. HPLC: retention time 11.970 min, purity 98.6% (254 nm).

*((R)-1-(((R)-2-hydroxy-3-phenylpropanoyl)-d-phenylalanyl)indoline-2-carbonyl)-l-arginine* (**12g**). Synthesized from **11g** (30 mg, 0.045 mmol) using the general procedure of ester hydrolysis, type B. White solid, yield: 21 mg (71%). ^1^H NMR (DMSO-*d*_6_, 400 MHz): δ 1.38–1.42 (m, 2H, -CHCH_2_CH_2_CH_2_NH-), 1.56–1.67 (m, 2H, -CHCH_2_CH_2_CH_2_NH-), 2.63–2.69 (m, 1H, -CH(OH)CH_2_Ph), 2.90–2.94 (m, 3H, -CH(OH)CH_2_Ph + -CHCH_2_CH_2_CH_2_NH-), 2.99–3.03 (m, 3H, -CHCH_2_Ph + -NHCHCH_2_-), 3.42 (dd, 1H, *J* = 16.5 Hz, *J* = 10.5 Hz, -NHCHCH_2_-), 3.92 (dd, 1H, *J* = 12.0 Hz, *J* = 5.9 Hz, -CHCH_2_CH_2_CH_2_NH-), 4.09 (dd, 1H, *J* = 8.1 Hz, *J* = 3.4 Hz, -CHCH_2_Ph), 4.68 (dd, 1H, *J* = 14.1 Hz, *J* = 6.3 Hz, -NHCHCH_2_-), 5.02 (dd, 1H, *J* = 10.9 Hz, *J* = 2.2 Hz, -CH(OH)CH_2_Ph), 7.00–7.05 (m, 3H, indoline Ar-H + -CHCH_2_Ph), 7.18–7.28 (m, 10H, indoline Ar-H + -CH(OH)CH_2_Ph + -CHCH_2_Ph), 7.69 (d, 1H, *J* = 8.0 Hz, -CONH-), 7.98 (d, 1H, *J* = 6.6 Hz, -CONH-), 8.03 (d, 1H, *J* = 8.0 Hz, indoline Ar-H), 8.36 (s, 1H, HCOO^−^), 8.92–8.96 (m, 1H, guanidine-NH) ppm. ^13^C NMR (DMSO-*d*_6_, 100 MHz): δ 174.42, 172.42, 168.82, 165.74, 157.93, 143.17, 138.90, 136.42, 130.58, 130.13, 130.02, 128.51, 128.38, 127.26, 127.04, 126.51, 125.09, 124.36, 116.86, 72.28, 60.90, 54.05, 52.23, 40.87, 40.76, 38.91, 35.00, 29.62, 25.55 ppm. HRMS for C_33_H_39_N_6_O_6_: calculated 615.2931; found 615.2943. IR(ATR) *ν*: 3320, 2964, 2777, 1740, 1617, 1481, 1451, 1412, 1352, 1199, 1132, 1004, 837, 800, 773, 765, 699, 632 cm^−1^. HPLC: retention time 11.951 min, purity 98.6% (254 nm).

*Methyl d-prolinate hydrochloride* (**12a**). d-proline (10.0 g; 86.8 mmol) was dissolved in 100 mL of absolute methanol and 12.3 mL of thionyl chloride (20.6 g; 173 mmol) was added dropwise at 0 °C. The reaction mixture was then stirred under the reflux for 3 h. The solvent was evaporated under the reduced pressure and oily product was washed with hexane to obtain 12.9 g of pure **15** (97%). ^1^H NMR (DMSO-*d*_6_, 400 MHz): δ 1.87–2.02 (m, 3H, Pro-H^3^, Pro-H^3^, Pro-H^4^), 2.22–2.31 (m, 1H, Pro-H^4^), 3.16–3.75 (m, 2H, Pro-H^5^), 3.77 (s, 3H, COOCH_3_), 4.38 (dd, *J* = 7.4 Hz, *J* = 8.5 Hz, 1H, Pro-H^2^) ppm; IR(ATR) *ν*: 765, 918, 1038, 1092, 1231, 1356, 1439, 1566, 1741, 2713, 2904 cm^−1^.

*Methyl ((R)-2-((tert-butoxycarbonyl)amino)-4-phenylbutanoyl)-d-prolinate* (**16**). Synthesized from aminoacid **14** (250 mg; 0.895 mmol) and **7a** (148 mg; 0.895 mmol) using the general coupling procedure type A. Oil, yield: 285 mg (81%). ^1^H NMR (DMSO-*d*_6_, 400 MHz): δ (ppm) 1.41 (s, 9H, (CH_3_)_3_C), 1.72–1.92 (m, 5H, Phe-β-CH_2_, Pro-H^4^, Pro-H^3^), 2.07–2.16 (m, 1H, Pro-H^3^), 2.55–2.57 (m, 2H, Ph-γ-CH_2_), 3.42–3.51 (m, 2H, Pro-H_5_), 3.59 (s, COOMe), 4.22 (4.89), (dd, *J* = 3.9 Hz, *J* = 8.8 Hz, 2H, Ph-α-CH; Pro-H^2^), 7.20–7.29 (m, 5H, -CH_2_CH_2_Ph), MS (ESI) *m*/*z* 377.3 (MH^+^, 100); IR(ATR) *ν*: 700, 750, 871, 1023, 1047, 1092, 1164, 1246, 1365, 1391, 1432, 1496, 1641, 1703, 1743, 2975, 3294 cm^−1^. HPLC (λ = 254 nm) R_t_ 13.19 min, 97.4%.

*((R)-2-((tert-butoxycarbonyl)amino)-4-phenylbutanoyl)-d-proline* (**17**). Synthesized from **16** (665 mg, 1.9 mmol) using the general procedure described in *6.7*. White solid, yield: 229 mg (81%). ^1^H NMR (DMSO-*d*_6_, 400 MHz): δ 1.39 (s, 9H, (CH_3_)_3_C), 1.73–1.91 (m, 5H, Phe-β-CH_2_, Pro-H^4^, Pro-H^3^), 2.05–2.18 (m, 1H, Pro-H^3^), 2.54–2.68 (m, 2H, Ph-γ-CH_2_), 3.50–3.51 (m, 2H, Pro-H^5^), 4.15 (dd, *J* = 3.4 Hz, *J* = 8.8 Hz, 2H, Ph-α-CH, Pro-H^2^), 4.23 (4.77) (dd, *J* = 8.6 Hz, *J* = 14.0 Hz, 1H, Ph-α-CH), 7.14–7.33 (m, 5H, -CH_2_CH_2_Ph) ppm; IR(ATR) *ν*: 669, 700, 739, 759, 886, 1021, 1041, 1156, 1192, 1244, 1273, 1338, 1364, 1388, 1455, 1520, 1609, 1700, 1716, 2883, 2951, 2980, 3023, 3280 cm^−1^. HPLC (λ = 254 nm) R_t_ 13.19 min, 93.4%

*Methyl N^2^-(((R)-2-((tert-butoxycarbonyl)amino)-4-phenylbutanoyl)-d-prolyl)-N^ω^-nitro-d-argininate* (**18**). Synthesized from **17** (160 mg; 0.439 mmol) and H-Arg(NO_2_)-OMe·HCl (118 mg; 0.439 mmol) using the general coupling procedure type A. White solid, yield: 259 mg (95%). ^1^H NMR (DMSO-*d*_6_, 400 MHz): δ 1.39 (s, 9H, (CH_3_)_3_C), 1.54–1.66 (m, 4H, Arg-β-CH_2_, Arg-γ-CH_2_), 1.73–1.89 (m, 5H, Phe-β-CH_2_, Pro-H^4^, Pro-H^3^), 1.95–2.07 (m, 1H, Pro-H^3^), 2.56–2.69 (m, 2H, Ph-γ-CH_2_), 3.15 (dd, *J* = 6.1 Hz, *J* = 12.3 Hz, 2H, Arg-δ-CH_2_), 3.52 (dd, *J* = 6.8 Hz, *J* = 13,3 Hz, 2H, Pro-H^5^), 3.61 (s, 3H, COOCH_3_), 4.12 (dd, *J* = 7.3 Hz, *J* = 13.5 Hz, 1H, Arg-α-CH) 4.21 (dd, *J* = 7.3 Hz, *J* = 12.9 Hz, 1H, Ph-α-CH), 4.28–4.32 (4.81–4.84) (m, 1H, Pro-H^2^), 7.13–7.31 (m, 5H, -CH_2_CH_2_Ph), 8.13 (d, *J* = 7.5 Hz, 1H, BocNHCH-), 8.52 (d, *J* = 7.8 Hz, 1H, -ProCONHCH-) ppm. ^13^C NMR (DMSO-*d*_6_, 100 MHz) δ 22.38, 24.64, 28.36, 29.09, 31.18, 31.40, 31.74, 34.25, 34.82, 46.50, 46.72, 51.45, 52.29, 58.96, 59.18, 76.74, 77.06, 77.38, 79.65, 79.84, 125.95, 126.07, 128.44, 128.48, 141.22, 155.44, 170.76, 172.40 ppm. IR(ATR) *ν*: 3305, 2952, 1741, 1622, 1529, 1435, 1391, 1365, 1251, 1161, 1047, 1024, 700, 728 cm^−1^. MS (ESI) *m*/*z* 592.3 (MH^+^, 100) HPLC: R_t_ 12.88 min, 91.3%. M.p. 93–94 °C.

*Methyl N^2^-(((R)-2-amino-4-phenylbutanoyl)-d-prolyl)-N^ω^-nitro-d-argininate* (**19**). To a solution of **18** (220 mg; 0.372 mmol) in 20 mL of dry methanol a dropwise of dry 1 M HCl (1.49 mL; 2.97 mmol) was added. The resulting mixture was stirred for an additional 15 min under an argon atmosphere. After 2 h, 1 M HCl was added (0.929 mL; 1.86 mmol) and it was left over the night at a room temperature. The next day, an additional 1 M HCl (0.929 mL; 1.86 mmol) was added and the reaction mixture was heated up to 35 °C and left over the night. The solvent was evaporated under the reduced pressure and flushed with argon to obtain the solid product. The yield of the reaction was 0.189 g (96%). ^1^H NMR (DMSO-*d6*, 400 MHz): δ 1.35–1.40 (m, 2H, Arg-γ-CH_2_), 1.66–1.78 (m, 2H, Arg-β-CH_2_), 1.82–1.92 (m, 3H, Phe-β-CH_2_, Pro-H^3^), 1.99–2.08 (m, 3H, Pro-H^3^, Pro-H^4^), 2.68–2.72 (m, 2H, Ph-γ-CH_2_), 3.36 (dd, *J* = 7.9 Hz, *J* = 16.8 Hz, 2H, Arg-δ-CH_2_), 3.46–3.52 (m, 2H, Pro-H^5^), 3.62 (s, 3H, COOCH_3_), 4.15–4.21 (m, 2H, Pro-H^2^, Ph-α-CH), 4.32 (4.84) (d, *J* = 9.8 Hz, 1H, Pro-H^2^), 7.22–7.34 (m, 5H, -CH_2_CH_2_Ph), 8.39–8.45 (m, 1H,-ProCONHCH-) 8.48 (d, *J* = 7.5 Hz, 2H, -NH_2_) ppm, MS (ESI) *m*/*z* 464.2 (MH^+^, 100); IR(ATR) *ν*: 626, 700, 1145, 1280, 1451, 1495, 1536, 1635, 1731, 2953, 3241 cm^−1^. HPLC (λ = 254 nm) R_t_ 10.15 min, 93.3%

*Methyl N^2^-(((R)-2-((R)-2-hydroxy-3-phenylpropanamido)-4-phenylbutanoyl)-d-prolyl)-N^ω^-nitro-d-argininate* (**20a**). Synthesized from **19** (98 mg; 0.186 mmol) and 3-phenyl-d-lactic acid (31 mg; 0.186 mmol) using the general coupling procedure type A. White solid, yield: 75 mg (63%). ^1^H NMR (DMSO-*d*_6_, 400 MHz): δ 1.47–1.66 (m, 4H, Arg-γ-CH_2_, Arg-β-CH_2_), 1.70–1.92 (m, 4H, Phe-β-CH_2_, Pro-H^4^), 1.97–2.08 (m, 1H, Pro-H^3^), 2.74–2.83 (m, 2H, Ph-γ-CH_2_), 2.97–3.04 (m, 2H, Phe-lactic-γ-CH_2_), 3.15 (dd, *J* = 6.1 Hz, *J* = 12.2 Hz, 2H, Arg-δ-CH_2_), 3.49–3.57 (m, 2H, Pro-H^5^), 3.61 (s, 3H, COOCH_3_), 4.08–4.23 (m. 1H, Ph-α-CH), 4.56–4.61 (4.79–4.83) (m, 1H, Pro-H^2^), 5.83 (d, 1H, Ph-lactic-OH) 7.16–7.31 (m, 10H, -CH_2_CH_2_Ph, Ph-lactic), 7.74 (7.82) (d, *J* = 8.3 Hz, 1H, -ProCONHCH-), 8.23 (8.56) (d, *J* = 7.5 Hz, 1H, Phe-lactic-NH) ppm. MS (ESI) *m*/*z* 640.3 (MH^+^, 100), IR(ATR) *ν*: 640, 698, 742, 845, 913, 1028, 1086, 1150, 1252, 1342, 1433, 1496, 1528, 1619, 1741, 3290 cm^−1^. HPLC (λ = 254 nm) R_t_ 12.51 min, 98.15%

*Methyl N^2^-(((2R)-2-((R,S)-2-hydroxy-3-(4-hydroxyphenyl)propanamido)-4-phenylbutanoyl)-d-prolyl)-N^ω^-nitro-d-argininate* (**20b**). Synthesized from **19** (91 mg; 0.172 mmol) and 2-hydroxy-3-(4-hydroxyphenyl)propanoic acid (35 mg; 0.172 mmol) using the general coupling procedure type C. White solid, yield: 20 mg (31%). ^1^H NMR (DMSO-*d*_6_, 400 MHz): δ 1.50–1.65 (m, 4H, Arg-γ-CH_2_, Arg-β-CH_2_), 1.70–1.92 (m, 4H, Phe-β-CH_2_, Pro-H^4^), 1.97–2.08 (m, 1H, Pro-H^3^), 2.54–2.57 (m, 2H, Ph-γ-CH_2_), 2.80–2.94 (m, 2H, Phe-lactic-γ-CH_2_), 3.11–3.21 (m, 2H,-Arg-δ-CH_2_), 3.48–3.57 (m, 2H, Pro-H^5^), 3.6 (s, 3H, -COOCH_3_), 4.05–4.15 (4.17–4.25) (m. 2H, Ph-α-CH, HO-Phelactic-α-CH), 4.27–4.34 (m, 1H, Ph-α-CH), 4.54–4.59 (4.79–4.82) (m, 1H, Pro-H^2^), 5.73 (5.53; 5.43) (d, *J* = 5.8 Hz, HO-Phe-lactic-OH), 6.98–7.31 (m, 9H, -CH_2_CH_2_Ph, OH-Phelactic), 7.71 (7.81; 7.97), (d, *J* = 8.3 Hz, 1H, OH-Ph-lactic-NH), 8.22 (8.51–8.57) (dd, *J* = 3.4 Hz, *J* = 7.6 Hz, 2H, -ProCONHCH-, OH-Ph-lactic-NH), 9.13 (t, *J* = 2.4 Hz, 1H, HO-Phelactic) ppm. MS (ESI) *m*/*z* 656.3 (MH^+^, 100). IR(ATR) *ν*: 620, 639, 699, 745, 1106, 1256, 1440, 1515, 1624, 1739, 2952, 3286 cm^−1^. HPLC (λ = 254 nm) R_t_ 11.31 min, 95.0%. M.p. 97–99 °C.

*Methyl ((R)-2-((R)-2-hydroxy-3-phenylpropanamido)-4-phenylbutanoyl)-d-prolyl-d-argininate* (**21a**). Synthesized from **20a** (55 mg; 0.085 mmol) using the general procedure of nitro group cleavage described in *6.10*. White solid, yield: 19 mg (37%). ^1^H NMR (DMSO-*d*_6_, 400 MHz): δ 1.51–1.89 (m, 8H, Arg-γ-CH_2_, Arg-β-CH_2_, 4H, Phe-β-CH_2_, Pro-H^4^), 1.97–2.11 (m, 2H, Pro-H^3^), 2.74–2.83 (m, 2H, Ph-γ-CH^2^), 3.02 (dd, *J* = 3.6 Hz, *J* = 13.7 Hz, 4H, Arg-δ-CH_2_, Pro-H^5^), 3.60 (s, 3H, COOCH_3_), 4.11–4.19 (4.55–4.61) (m, 2H, Ph-α-CH, Arg-α-CH), 4.31 (4.83) (dd, *J* = 4.4 Hz, *J* = 6.6 Hz, (m, 1H, Pro-H^2^), 6.01–6.26 (m, 2H, Arg-NH_2_), 7.13–7.31 (m, 10H, -CH_2_CH_2_Ph; Ph-lactic), 7.82 (8.90) (dd, *J* = 9.5 Hz, *J* = 20.7 Hz, 1H, -ProCONHCH-), 9.16 (9.30) (s, 1H, Phe-lactic-NH) ppm, MS (ESI) 595.3 (MH^+^, 100), IR(ATR) *ν*: 699, 772, 1088, 1200, 1353, 1390, 1452, 1589, 1739, 2828, 2925, 3962, 3280 cm^−1^. HPLC (λ = 254 nm) R_t_ 15.98 min, 96.9%. M.p. 69–70 °C.

*Methyl ((R)-2-((R,S)-2-hydroxy-3-(4-hydroxyphenyl)propanamido)-4-phenylbutanoyl)-d-prolyl-d-argininate* (**21b**). Synthesized from **20b** (62.0 mg; 0.094 mmol) using the general procedure of nitro group cleavage described in *6.10*. White solid, yield: 20 mg (31%). ^1^H NMR (DMSO-*d*_6_, 400 MHz): δ 1.52–1.90 (m, 8H, Arg-γ-CH_2_, Arg-β-CH_2_, 4H, Phe-β-CH_2_, Pro-H4), 1.97–2.10 (m, 2H, Pro-H^3^), 2.72–2.81 (m, 2H, Ph-γ-CH_2_), 3.01 (dd, *J* = 3.6 Hz, *J* = 13.6 Hz, 4H, Arg-δ-CH_2_, Pro-H^5^), 3.59 (s, 3H, COOCH_3_), 4.11–4.19 (4.55–4.61) (m, 2H, Ph-α-CH, Arg-α-CH), 4.31 (4.83) (dd, *J* = 4.4 Hz, *J* = 6.6 Hz, (m, 1H, Pro-H^2^), 6.01–6.26 (m, 2H, Arg-NH_2_), 7.13–7.31 (m, 9H, -CH_2_CH_2_Ph; -CH(OH)CH_2_Ph(OH)), 7.81 (8.99) (dd, *J* = 9.4 Hz, *J* = 20.6 Hz, 1H, -ProCONHCH-), 8.91 (8.93) (s, 1H, -CH(OH)CH_2_Ph(OH)) 9.16 (9.30) (s, 1H, Phe-lactic-NH) ppm, IR(ATR) *ν*: 699, 814, 1018, 1104, 1200, 1352, 1444, 1513, 1608, 1648, 1730, 3149 cm^−1^. MS (ESI) *m*/*z* 611.3 (MH^+^, 100). HPLC ret. time 10.63 min and 10.67, 85.09%. M.p. 105–106 °C.

*(R)-2-azido-4-methylpentanoic acid* (**23**). Synthesized from d-leucine (1.20 g; 9.15 mmol) using the general procedure described in *6.4*. Brown oil, yield: 1.03 g (71%) and was used without additional purification and characterisation.

*Methyl (R)-1-((R)-2-azido-4-methylpentanoyl)indoline-2-carboxylate* (**24a**). Synthesized from **23** (1.26 g, 6 mmol) and **5** (1.07 g, 5 mmol) using the general procedure described in *6.6*. Colourless oil, yield: 0.71 g (40%). ^1^H NMR (DMSO-*d*_6_, 400 MHz): δ 0.93 (dd, *J* = 6.5 Hz, *J* = 22.3 Hz, 6H, leucine-δ-CH_3_, leucine-δ-CH_3_), 1.53–1.59 (m, 2H, leucine-γ-CH_2_), 1.68–1.84 (m, 2H, leucine-β-CH_2_), 3.27–3.34 (m, 1H, indoline-H^3^), 3.62–2.70 (m, 1H, indoline-H^3^), 4.01(dd, *J* = 3.6 Hz, *J* = 9.6 Hz, 1H, indoline-H^2^), 5.6 (d, *J* = 10.5 Hz, 1H, leucine-α-CH), 7.10 (dd, *J* = 4.2 Hz, *J* = 11.5 Hz, 1H, indoline Ar-H), 7.22–7.30 (m, 2H, indoline Ar-H;), 8.08 (d, *J* = 8.0 Hz, 1H, indoline Ar-H) ppm. HPLC (λ = 254 nm) R_t_ 16.15 min, 99.7%. M.p. 67–68 °C.

*Methyl (S)-1-((S)-2-azido-4-methylpentanoyl)indoline-2-carboxylate* (**24b**). Synthesized from **23** (1.26 g, 6 mmol) and **5** (1.07 g, 5 mmol) using the general procedure described in *6.6*. Colourless oil, yield: 0.69 g (43%). ^1^H NMR (DMSO-*d*_6_, 400 MHz): δ 0.93 (dd, *J* = 6.5 Hz, *J* = 22.3 Hz, 6H, leucine-δ-CH_3_, leucine-δ-CH_3_), 1.53–1.59 (m, 2H, leucine-γ-CH_2_), 1.68–1.84 (m, 2H, leucine-β-CH_2_), 3.27–3.34 (m, 1H, indoline-H^3^), 3.62–2.70 (m, 1H, indoline-H^3^), 4.01(dd, *J* = 3.6 Hz, *J* = 9.6 Hz, 1H, indoline-H^2^), 5.6 (d, *J* = 10.5 Hz, 1H, leucine-α-CH), 7.10 (dd, *J* = 4.2 Hz, *J* = 11.5 Hz, 1H, indoline Ar-H), 7.22–7.30 (m, 2H, indoline Ar-H), 8.08 (d, *J* = 8.0 Hz, 1H, indoline Ar-H) ppm. HPLC (λ = 254 nm) R_t_ 16.15 min, 99.7%. M.p. 67–68 °C.

*(R)-1-((R)-2-azido-4-methylpentanoyl)indoline-2-carboxylic acid* (**25a**). Synthesized from **24a** (254 mg; 0.803 mmol) using the general procedure of ester hydrolysis (*6.6.*) White solid, yield: 213 mg (87%). ^1^H NMR (DMSO-*d*_6_, 400 MHz): δ 0.96 (dd, *J* = 6.1 Hz, *J* = 12.8 Hz, 6H, leucine-δ-CH_3_, leucine-δ-CH_3_), 1.68–1.81 (m, 4H, leucine-γ-CH_2_, leucine-β-CH_2_), 3.2–3.4 (d, *J* = 16.8 Hz,1H indoline-H^3^), 3.64 (dd, *J* = 10.7 Hz, *J* = 16.6 Hz, 1H, indoline-H^3^), 3.81–3.85 (dd, *J* = 3.8 Hz, *J* = 9.7 Hz, 1H, indoline-H^2^), 5.06 (dd, *J* = 2.3 Hz, *J* = 10.8 Hz, 1H, leucine-α-CH), 7.08 (dt, *J* = 1.0 Hz, *J* = 7.4 Hz, *J* = 7.5 Hz, 1H, indoline Ar-H), 7.21–7.29 (m, 2H, indoline Ar-H), 8.12 (d, i = 8.00 Hz, 1H, indoline Ar-H) ppm. IR(ATR) *ν*: 752, 1120, 1166, 1208, 1233, 1304, 1405, 1463, 1481, 1596, 1655, 1724, 2109, 2871, 2958 cm^−1^. HPLC (λ = 254 nm) R_t_ 17.25 min., 97.4%

*(S)-1-((R)-2-azido-4-methylpentanoyl)indoline-2-carboxylic acid* (**25b**). Synthesized from **24b** (201 mg; 0.635 mmol) using the general procedure of ester hydrolysis (*6.6.*). White solid, yield: 146 mg (87%). 1H NMR (DMSO-*d*_6_, 400 MHz): δ 0.93 (dd, *J* = 6.3 Hz, *J* = 15.6 Hz, 6H, leucine-δ-CH_3_, leucine-δ-CH_3_), 1.61–1.84 (m, 4H, leucine-γ-CH_2_, leucine-β-CH_2_), 3.3 (d, *J* = 16.8 Hz,1H indoline-H^3^), 3.61 (dd, *J* = 10.7 Hz, *J* = 16.6 Hz, 1H, indoline-H^3^), 3.97 (dd, *J* = 3.8 Hz, *J* = 9.7 Hz, 1H, indoline-H^2^), 5.40 (dd, *J* = 5.0 Hz, *J* = 6.2 Hz, 1H, leucine-α-CH), 7.09 (dt, *J* = 1.0 Hz, *J* = 7.5 Hz, 1H, indoline Ar-H), 7.21–7.29 (m, 2H, indoline Ar-H), 8.1 (d, *J* = 7.9 Hz, 1H, indoline Ar-H) ppm. IR(ATR) *ν*: 634, 684, 707, 757, 832, 870, 912, 1022, 1093, 1119, 1181, 1216, 1253, 1265, 1355, 1402, 1429, 1461, 1732, 2114, 2869, 2955, 3072 cm^−1^. HPLC (λ = 254 nm) R_t_ 14.23 min, 98.6%.

*Methyl N^2^-((R)-1-((R)-2-azido-4-methylpentanoyl)indoline-2-carbonyl)-N^ω^-nitro-l-argininate* (**26a**). Synthesized from **25b** (213 mg; 0.705 mmol) using the general coupling procedure type A. White solid, yield: 255 mg (70%). 1H NMR (DMSO-*d*_6_, 400 MHz): δ 1.00 (dd, *J* = 6.5 Hz, *J* = 9.9 Hz, 6H, leucine-δ-CH_3_, leucine-δ-CH_3_), 1.53–1.82 (m, 8H, Arg-β-CH_2_, Arg-γ-CH_2_, leucine-γ-CH_2_, leucine-β-CH_2_), 3.18 (dd, *J* = 5.9 Hz, *J* = 13.2 Hz, 2H, Arg-δ-CH_2_), 3.63 (s, 3H, COOCH_3_), 3.66–3.76 (s, 2H, indoline-H^3^), 4.19–4.25 (m, 1H, indoline-H^2^), 4.95 (dd, *J* = 3.2 Hz, *J* = 11.1 Hz, 1H, leucine-α-CH), 7.05 (dt, *J* = 0.77 Hz, *J* = 7.42 Hz, 1H, indoline Ar-H), 7.22 (dd, *J* = 7.4 Hz, *J* = 8.2 Hz, 2H, indoline Ar-H), 8.13 (d, *J* = 8.2 Hz, 1H, indoline Ar-H), 8.89 (d, *J* = 6.8 Hz, 1H, indoline-CONHCH-) ppm. IR(ATR) *ν*: 754, 914, 1021, 1149, 1255, 1405, 1462, 1481, 1537, 1597, 1626, 1742, 2112, 2870, 2956, 3299 cm^−1^. HPLC (λ = 254 nm) R_t_ 13.30 min, 98.3%.

*Methyl N^2^-((S)-1-((R)-2-azido-4-methylpentanoyl)indoline-2-carbonyl)-N^ω^-nitro-l-argininate* (**26b**). Synthesized from **25b** (146 mg; 0.483 mmol) using the general coupling procedure type A. White solid, yield: 152 mg (61%). 1H NMR (DMSO-*d*_6_, 400 MHz): δ 0.88 (dd, *J* = 6.5 Hz, *J* = 24.0 Hz, 6H, leucine-δ-CH_3_, leucine-δ-CH_3_), 1.50–1.80 (m, 8H, Arg-β-CH_2_, Arg-γ-CH_2_, leucine-γ-CH_2_, leucine-β-CH_2_), 3.13–3.21 (m, 2H, Arg-δ-CH_2_), 3.61 (s, 5H, COOCH_3_, indoline-H^3^), 4.04–4.08 (m, 1H, 1H, Arg-α-CH), 4.14–4.21 (m, 1H, indoline-H^2^), 5.25 (dd, *J* = 3.4 Hz, *J* = 11.5 Hz, 1H, leucine-α-CH), 7.07 (t, *J* = 7.93 Hz, 1H, indoline Ar-H), 7.20–7.29 (m, 2H, indoline Ar-H), 8.09 (d, *J* = 9.0 Hz, 1H, indoline Ar-H), 8.81 (d, *J* = 7.4 Hz, 1H, indoline-CONHCH-) ppm. IR(ATR) *ν*: 659, 755, 1003, 1100, 1150, 1209, 1254, 1408, 1462, 1481, 1536, 1597, 1656, 1742, 2104, 2870, 2955, 3295 cm^−1^. HPLC (λ = 254 nm) R_t_ 13.27 min, 98.1%.

*Methyl N^2^-((R)-1-(d-leucyl)indoline-2-carbonyl)-N^ω^-nitro-l-argininate* (**27a**). Synthesized from **26a** (272 mg; 0,553 mmol) using the general procedure of azido group reduction (*6.9.*). Crude product (187 mg, 69%) was used further without purification.

*Methyl N^2^-((S)-1-(d-leucyl)indoline-2-carbonyl)-N^ω^-nitro-l-argininate* (**27b**). Synthesized from **26b** (390 mg; 0.753 mmol) using the general procedure of azido group reduction (*6.9.*). Crude product (185 mg, 50%) was used further without purification.

*Methyl N^2^-((R)-1-(((R)-2-hydroxy-3-phenylpropanoyl)-d-leucyl)indoline-2-carbonyl)-N^ω^-nitro-l-argininate* (**28a**). Synthesized from **27a** (187 mg; 0.380 mmol) and 3-phenyl-d-lactic acid (64.5 mg; 0.380 mmol) using the general coupling procedure type C. White solid, yield: 207 mg (83%). ^1^H NMR (DMSO-*d6*, 400 MHz): δ 0.82 (d, *J* = 6.3 Hz, 3H, leucine-δ-CH_3_), 0.97 (d, *J* = 6.1, 3H, leucine-δ-CH_3_),1.3–1.81 (m, 8H, Arg-β-CH_2_, Arg-γ-CH_2_, leucine-γ-CH_2_, leucine-β-CH_2_), 2.73–2.79 (m, 2H, Phe-lactic-γ-CH_2_), 3.05–3.20 (m, 2H, Arg-δ-CH_2_), 3.65 (s, 3H, COOCH_3_), 3.69–3.78 (m, 2H, indoline-H^3^), 4.20–4.29 (m, 1H, 1H, Arg-α-CH), 4.41 (t, *J* = 10.3 Hz, 1H, indoline-H^2^), 4.97 (dd, *J* = 10.8 Hz, 1H, leucine-α-CH), 5.93 (d, *J* = 5.7 Hz, 1H, Phe-lactic-β-CH_2_), 7.01–7.09 (m, 1H, indoline Ar-H), 7.17–7.34 (m, 7H, indoline Ar-H, Phe-lactic-), 7.6 (d, *J* = 8.8 Hz, 1H, Phe-lactic-NH-), 8.09 (d, *J* = 8.5 Hz, 1H, indoline Ar-H), 8.79 (d, *J* = 9.4 Hz, 1H, indoline-CONHCH-) ppm, MS (ESI) *m*/*z* 640.3 (MH+, 100), IR(ATR) *ν*: 615, 640, 700, 7541, 1017, 1085, 1155, 1259, 1407, 1463, 1481, 1525, 1597, 1628, 1739, 2358, 2870, 2924, 2954, 3104, 3327, 3389, 3435, 3598 cm^−1^. HPLC (λ = 254 nm) R_t_ 13.29 min, 94.3%. M.p. 106–107 °C

*Methyl N^2^-((S)-1-(((R)-2-hydroxy-3-phenylpropanoyl)-d-leucyl)indoline-2-carbonyl)-N^ω^-nitro-l-argininate* (**28b**). Synthesized from **27b** (185 mg; 0.376 mmol) and 3-phenyl-d-lactic acid (64.5 mg; 0.380 mmol) using the general coupling procedure type C. White solid, yield: 99 mg (41%). ^1^H NMR (DMSO-*d*_6_, 400 MHz): δ 0.70 (d, *J* = 6.4 Hz, 3H, leucine-δ-CH_3_), 0.81 (d, *J* = 6.5, 3H, leucine-δ-CH_3_), 1.21–1.40 (m, 4H, Arg-β-CH_2_, Arg-γ-CH_2_), 1.57–1.81 (m, 4H, leucine-γ-CH_2_, leucine-β-CH_2_), 2.76 (dd, *J* = Hz, 6.7 Hz, *J* = 14.1 Hz, 2H, Phe-lactic-γ-CH^2^), 3.13–3.25 (m, 2H, Arg-δ-CH_2_), 3.39–3.48 (m, 1H, indoline-H^3^), 3.61 (s, 3H, COOCH_3_), 4.13–4.20 (m, 1H, Arg-α-CH), 4.27–4.33 (m, 1H, indoline-H^2^), 5.69 (dd, *J* = 5.5 Hz, *J* = 13.4 Hz, 2H, leucine-α-CH, Phe-lactic-β-CH_2_), 7.01–7.07 (t, *J* = 7.93 Hz, 1H, indoline Ar-H), 7.11–7.26 (m, 7H, indoline Ar-H, Phe-lactic-), 7.82 (d, *J* = 6.2 Hz, 1H, Phe-lactic-NH-), 8.10 (d, *J* = 8.4 Hz, 1H, indoline Ar-H), 8.90 (d, *J* = 8.9 Hz, 1H, indoline-CONHCH-) ppm. MS (ESI) *m*/*z* 640.3 (MH^+^, 100). IR(ATR) *ν*: 616, 699, 749, 1003, 1086, 1152, 1209, 1256, 1342, 1418, 1481, 1534, 1595, 1630, 1740, 2954, 3287, 3675, 3853 cm^−1^. HPLC (λ = 254 nm) R_t_ 12.77 min, 91.5%

*Methyl ((S)-1-(((R)-2-hydroxy-3-phenylpropanoyl)-d-leucyl)indoline-2-carbonyl)-l-argininate* (**29a**). Synthesized from **28a** (70 mg; 0.109 mmol) using the general procedure of nitro group cleavage described in *6.10*. White solid, yield: 45 mg (69%). ^1^H NMR (DMSO-*d*_6_, 400 MHz): δ 0.72 (d, *J* = 6.2 Hz, 3H, leucine-δ-CH_3_), 0.82 (d, *J* = 6.5, 3H, leucine-δ-CH_3_), 1.22–1.37 (m, 8H, Arg-β-CH_2_, Arg-γ-CH_2_, leucine-γ-CH_2_, leucine-β-CH_2_), 1.72–1.82 (dd, *J* = 6.7 Hz, *J* = 14.1 Hz, 2H, Phe-lactic-γ-CH_2_), 2.87–3.01(m, 2H, Arg-δ-CH_2_), 3.07–3.67 (m, 1H, indoline-H^3^), 3.61 (s, 3H, COOCH_3_), 4.00–4.18 (m, 1H, Arg-α-CH), 4.24–4.28 (m, 1H, indoline-H^2^), 5.60 (dd, *J* = 3.9 Hz, *J* = 9.5 Hz, 2H, leucine-α-CH, Phe-lactic-β-CH_2_), 7.03 (t, *J* = 7.6 Hz, 1H, indoline Ar-H), 7.11–7.60 (m, 7H, indoline Ar-H, Phe-lactic-), 7.86 (d, *J* = 8.7 Hz, 1H, Phe-lactic-NH-), 8.01–8.11 (d, *J* = 8.4 Hz, 3H, indoline-Ar-H, Arg-NH_2_), 9.0 (d, *J* = 7.6 Hz, 1H, indoline-CONHCH-) ppm. MS (ESI) *m*/*z* 595.3 (MH^+^, 100). IR(ATR) *ν*: 621, 753, 911, 1001, 1065, 1147, 1241, 1346, 1402, 1433, 1462, 1531, 1594, 1620, 1656, 1742, 2104, 2133, 2954, 3236 cm^−1^. HPLC (λ = 254 nm) R_t_ 11.82 min, 96.0%.

*Methyl ((R)-1-(((R)-2-hydroxy-3-phenylpropanoyl)-d-leucyl)indoline-2-carbonyl)-l-argininate* (**29b**). Synthesized from **28b** (185 mg; 0.282 mmol) using the general procedure of nitro group cleavage described in *6.10*. White solid, yield: 77 mg (46%). ^1^H NMR (DMSO-*d*_6_, 400 MHz): δ 0.82 (s, 3H, leucine-δ-CH_3_), 0.98 (s, 3H, leucine-δ-CH_3_), 1.19 (t, *J* = 7.8 Hz, 1H, Arg-β-CH_2_), 1.38–1.79 (m, 7H, Arg-γ-CH_2_, leucine-γ-CH_2_, leucine-β-CH_2_), 2.73–2.76 (m, 2H, Phe-lactic-γ-CH_2_), 2.98–3.16 (m, 2H, Arg-δ-CH_2_), 3.40–3.71 (m, 5H, indoline-H^3^, COOCH_3_), 4.03–4.21 (m, 1H, Arg-α-CH), 4.99(d, *J* = 10.8 Hz, 1H, indoline-H^2^), 7.12–7.30 (m, 1H, indoline Ar-H), 7.63–8.01 (m, 8H, indoline Ar-H, Phe-lactic-), 8.44 (s, 1H, Phe-lactic-NH-), 9.01 (d, *J* = 27.4 Hz, 1H, Arg-NH_2_) ppm. ^13^C NMR (DMSO-*d*_6_, 100 MHz): δ 14.07, 20.75, 21.16, 23.47, 23.95, 24.98, 28.08, 34.54, 42.68, 48.99, 51.96, 52.06, 59.74, 69.27, 71.83, 116.27, 123.79, 124.57, 125.95, 127.15, 127.81, 129.60, 129.85, 138.13, 143.00, 157.46, 166.42, 171.06, 171,78, 171.98 ppm. MS (ESI) *m*/*z* 595.3 (MH^+^, 100) IR(ATR) *ν*: 633, 699, 757, 833, 1092, 1119, 1181, 1216, 1254, 1352, 1423, 1462, 1481, 1595, 1644, 1732, 2115, 2868, 2955, 3070, 3181, 3339, 3477 cm^−1^. HPLC (λ = 254 nm) R_t_ 12.21 min, 95.3%. M.p. 112–113 °C

## 7. Biological Evaluation-Enzyme Assay for Inhibition of Serine Proteases

The enzyme amidolytic method for determining inhibition was based on the spectrophotometric determination of absorbance of the product formed after amide bond cleavage of a chromogenic substrate in the presence of the enzyme. Ki, which is a quantitative measure of inhibitor potency, was determined from the kinetics of substrate hydrolysis with and without the addition of the inhibitor. Measurements (spectrophotometer, BioTek Synergy H4, BioTek Instruments, Winooski, VT, USA) were performed in 96-well microtiter plates with a final volume of 200 µL. Thrombin (Thrombin from human plasma, Sigma, Taufkirchen, Germany) was tested at a final concentration of 0.5 NIH E/mL with the substrate S-2238 (*H*-d-Phenylalanyl-*l*-pipecolyl-*l*-arginine-p-nitroaniline dihydrochloride, Chromogenix AB, Mondal, Sweden) at 20 and 40 mM final concentration, and factor Xa (Factor Xa from bovine plasma, Chromogenix) at the final concentration of 1 mBAEE E/mL with the substrate S-2222 (*N*-Benzoyl-*l*-isoleucyl-*l*-glutamyl-glycyl-*l*-arginine-*p*-nitroaniline hydrochloride and its methyl ester, Chromogenix) at 100 and 200 mM final concentrations. Trypsin (Trypsin from bovine pancreas, Sigma) was assayed at a final concentration of 0.5 nkat/mL using the substrate S-2222 (Chromogenix) at 50 and 100 mM final concentrations. Inhibitors were dissolved in DMSO (concentration of stock solutions, 10 mmol/L) and diluted with distilled water to concentrations from 0.5 to 100 mM. Reaction rates were measured in the presence and the absence of the inhibitor. Then, 50 µL HBSA buffer, 50 µL solution of each inhibitor concentration (or of HBSA buffer in case of measurement without inhibitor), and 50 µL of enzyme solution were pipetted into the microtiter wells. The plate was incubated for 15 min at 25 °C and then 50 mL of chromogenic substrate was added. Absorbance at 405 nm at 25 °C was measured every 10 s. Measurements were carried out in triplicate with three concentrations of the inhibitor and two concentrations of the substrate. For every combination of concentrations, K_i_ was calculated from the change of absorbance in the initial, linear part of the curve according to the method of Cheng and Prusoff and the final result was given as their average value. Dabigatran (thrombin inhibitory activity, K_i_ = 6.3 ± 1.1 nM) was used as the control.

## Figures and Tables

**Table 1:** Thrombin inhibitory activity of selected spumigins.

Spumigins	*Thrombin Inhibition* EC_50_
spumigin A (racemate)	7.5 µM [2]
spumigin A	2.1 µM [5]
spumigin J	4.9 µM [5]

**Table 2:** Thrombin, factor Xa and trypsin inhibitory activity of prepared spumigins analogues.

Structure	Compound	K_i_ Thrombin	K_i_ Factor Xa	K_i_ Trypsin
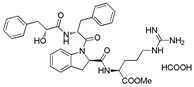	**11a**	52 ± 10 µM	49 ± 4 µM	7.9 ± 3.5 µM
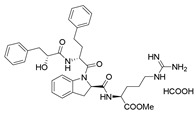	**11b**	44 ± 7 µM	39 ± 6 µM	2.8 ± 0.4 µM
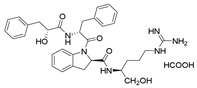	**11c**	92 ± 20 µM	54 ± 3 µM	54 ± 1 µM
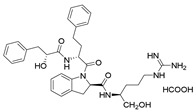	**11d**	11 ± 4 µM	47 ± 6 µM	29 ± 2 µM
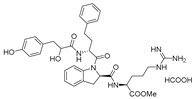	**11e**	30 ± 12 µM	48 ± 5 µM	3.9 ± 0.3 µM
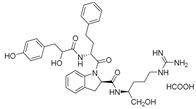	**11f**	20 ± 3 µM	76 ± 3 µM	14 ± 1 µM
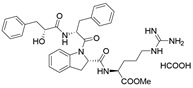	**11g**	50 ± 9 µM	47 ± 8 µM	126 ± 14 µM
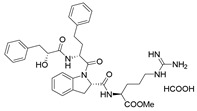	**11h**	24 ± 8 µM	35 ± 9 µM	58 ± 17 µM
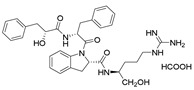	**11i**	170 ± 58 µM	51 ± 4 µM	50 ± 3 µM
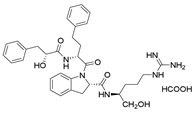	**11j**	187 ± 79 µM	41 ± 9 µM	693 ± 159 µM
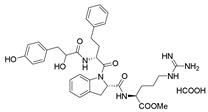	**11k**	34 ± 10 µM	57 ± 8 µM	72 ± 6 µM
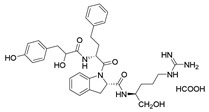	**11l**	186 ± 48 µM	62 ± 4 µM	62 ± 3 µM
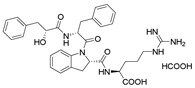	**12g**	98 ± 26 µM	69 ± 1 µM	0.28 ± 0.04 µM
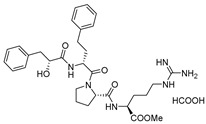	**21a**	3.8 ± 0.4 µM (IC_50_ = 33 ± 3 µM)	18 ± 4 µM (IC_50_ = 29 ± 6 µM)	1.4 ± 0.2 µM (IC_50_ = 4.2 ± 0.6 µM)
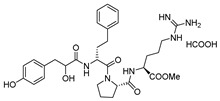	**21b**	3.9 ± 0.4 µM	29 ± 5 µM	0.92 ± 0.08 µM
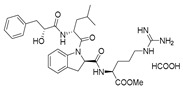	**29a**	5.6 ± 0.3 µM	30 ± 2 µM	4.8 ± 0.7 µM
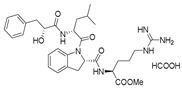	**29b**	129 ± 13 µM	65 ± 2 µM	134 ± 42 µM

## Supplementary Materials

The following are available online at http://www.mdpi.com/1660-3397/16/11/413/s1, Spectral data of the synthetic compounds.
