# Supplementary material for: Synthesis and Evaluation of Spumigin Analogues Library with Thrombin Inhibitory Activity
Source: Mar Drugs. 2018 Oct 27;16(11):413. doi: 10.3390/md16110413 (PMC6266488; doi:10.3390/md16110413)
Supplement: Supplementary file 1 [file marinedrugs-16-00413-s001.pdf]

Supplementary Materials

Synthesis and evaluation of spumigin analogues library with thrombin inhibitory activity

Aleš Žula [1], Izabela Będziak [1,2], Danijel Kikelj [1], Janez Ilaš [1,*]

[1] Faculty of Pharmacy, University of Ljubljana, Aškerčeva 7, 1000 Ljubljana, Slovenia
[2] Faculty of Pharmacy, The Poznan University of Medical Sciences, ul. Fredry 10, 61-701 Poznań, Poland
* Correspondence: janez.ilas@ffa.uni-lj.si; +386 1 4769 576

(R)-2-Azido-4-phenylbutanoic acid (**2b**)

Synthesized from **1b** (10 mmol) using the general procedure described in **6.4.** Brown oil, yield: 1.48 g (72%). ^{1}H NMR (DMSO-d_6, 400 MHz): δ 1.86-1.95 (m, 1H, -CH$\underline{\text{CH}_2}$CH$_2$Ph), 2.00-2.09 (m, 1H, -CH$\underline{\text{CH}_2}$CH$_2$Ph), 2.65-2.69 (m, 2H, -CHCH$_2$$\underline{\text{CH}_2}$Ph), 4.08 (dd, 1H, *J* = 8.8 Hz, *J* = 4.6 Hz, -$\underline{\text{CH}}$CH$_2$CH$_2$Ph), 7.19-7.23 (m, 3H, -CHCH$_2$CH$_2$$\underline{\text{Ph}}$), 7.28-7.33 (m, 2H, -CHCH$_2$CH$_2$$\underline{\text{Ph}}$), 13.40 (bs, 1H, -COO$\underline{\text{H}}$) ppm. ^{13}C NMR (DMSO-$d_6$, 100 MHz): δ 172.19, 141.03, 128.92, 128.79, 126.57, 61.26, 33.03, 31.77 ppm. IR(ATR): ν = 3028, 2929, 2104, 1713, 1497, 1454, 1419, 1227, 1030, 910, 748, 698 cm^{-1}.

Methyl (*R*)-1-((*R*)-2-azido-4-phenylbutanoyl)indoline-2-carboxylate (**6b**)

Synthesized from **3b** (1.26 g, 6 mmol) and **5** (1.07 g, 5 mmol) using the general procedure described in **6.6.** Colourless oil, yield: 0.75 g (41%). ^{1}H NMR (DMSO-d_6, 400 MHz): δ 2.06-2.13 (m, 2H, -CH$\underline{\text{CH}_2}$CH$_2$Ph), 2.62-2.81 (m, 2H, -CHCH$_2$$\underline{\text{CH}_2}$Ph), 3.24-3.30 (m, 1H, -NHCH$\underline{\text{CH}_2}$-), 3.62-3.68 (m, 4H, -COO$\underline{\text{Me}}$ + -NHCH$\underline{\text{CH}_2}$-), 3.95 (dd, 1H, *J* = 8.3 Hz, *J* = 5.2 Hz, -$\underline{\text{CH}}$CH$_2$CH$_2$Ph), 5.38 (dd, 1H, *J* = 10.6 Hz, *J* = 1.5 Hz, -NH$\underline{\text{CH}}$CH$_2$-), 7.10 (t, 1H, *J* = 7.4 Hz, indoline Ar-$\underline{\text{H}}$), 7.22-7.33 (m, 7H, indoline Ar-$\underline{\text{H}}$), 8.09 (d, 1H, *J* = 8.0 Hz, indoline Ar-$\underline{\text{H}}$) ppm. HRMS for $C_{20}H_{21}N_4O_3$: calculated 365.1614; found 365.1616. IR(ATR): ν = 3331, 3025, 2954, 2110, 1734, 1664, 1600, 1479, 1462, 1410, 1338, 1309, 1235, 1207, 1004, 936, 858, 748, 698, 668 cm^{-1}.

Methyl (*S*)-1-((*R*)-2-azido-3-phenylpropanoyl)indoline-2-carboxylate (**6c**)

Synthesized from **3a** (1.26 g, 6 mmol) and **5** (1.07 g, 5 mmol) using the general procedure described in **6.6.** Colourless oil, yield: 0.68 g (39%). $[\alpha]^{25}_D$ -0.60 (c 0.229, MeOH), ^{1}H NMR (MeOH-d_4, 400 MHz): δ 2.95 (dd, 1H, *J* = 16.3 Hz, *J* = 10.3 Hz, -CH$\underline{\text{CH}_2}$Ph), 3.14-3.21 (m, 3H, -NHCH$\underline{\text{CH}_2}$- + -CH$\underline{\text{CH}_2}$Ph), 3.77 (s, 3H, -COO$\underline{\text{Me}}$), 4.27 (t, 1H, *J* = 7.8 Hz, -$\underline{\text{CH}}$CH$_2$Ph), 4.51 (dd, 1H, *J* = 10.3, *J* = 1.9 Hz, -NH$\underline{\text{CH}}$CH$_2$-), 7.07 (dt, 1H, *J* = 7.4 Hz, *J* = 0.8 Hz, indoline Ar-$\underline{\text{H}}$), 7.16 (d, 1H, *J* = 7.3 Hz, indoline Ar-$\underline{\text{H}}$), 7.21-7.29 (m, 6H, -CHCH$_2$$\underline{\text{Ph}}$ + indoline Ar-$\underline{\text{H}}$), 8.19 (d, 1H, *J* = 8.1 Hz, indoline Ar-$\underline{\text{H}}$) ppm.^{13}C NMR (MeOH-d_4, 100 MHz): δ 172.73, 170.83, 142.76, 137.05, 130.95, 130.59, 129.83, 128.67, 128.45, 126.11, 125.62, 118.64, 63.03, 61.79, 53.66, 39.52, 33.56 ppm. HRMS for $C_{19}H_{19}N_4O_3$: calculated 351.1457; found 351.1459. IR(ATR): ν = 3330, 3028, 2954, 2105, 1736, 1662, 1599, 1482, 1463, 1406, 1308, 1230, 1205, 1665, 1081, 1022, 1006, 931, 749, 698, 666 cm^{-1}.

Methyl (*S*)-1-((*R*)-2-azido-4-phenylbutanoyl)indoline-2-carboxylate (**6d**)

Synthesized from **3b** (1.26 g, 6 mmol) and **5** (1.07 g, 5 mmol) using the general procedure described in **6.6.** Colourless oil, yield: 0.67 g (37%). ^{1}H NMR (DMSO-d_6, 400 MHz): δ 1.97-2.06 (m, 1H, -CH$\underline{\text{CH}_2}$CH$_2$Ph), 2.18-2.26 (m, 1H, -CH$\underline{\text{CH}_2}$CH$_2$Ph), 2.67-2.84 (m, 2H, -CHCH$_2$$\underline{\text{CH}_2}$Ph), 3.25-3.35 (m, 1H, -NHCH$\underline{\text{CH}_2}$-), 3.60-3.66 (m, 4H, -COO$\underline{\text{Me}}$ + -NHCH$\underline{\text{CH}_2}$-), 3.92 (dd, 1H, *J* = 9.3 Hz, *J* =4.5 Hz, -$\underline{\text{CH}}$CH$_2$CH$_2$Ph), 5.15 (dd, 1H, *J* = 10.7 Hz, *J* = 1.7 Hz, -NH$\underline{\text{CH}}$CH$_2$-), 7.09 (dt, 1H, *J* = 7.5 Hz, *J* = 0.8 Hz, indoline Ar-$\underline{\text{H}}$), 7.19-7.33 (m, 7H, indoline Ar-$\underline{\text{H}}$), 8.13 (d, 1H, *J* = 7.9 Hz, indoline Ar-$\underline{\text{H}}$) ppm. HRMS for $C_{20}H_{21}N_4O_3$: calculated 365.1614; found 365.1616; found. IR(ATR): ν = 3331, 3024, 2953, 2105, 1741, 1664, 1600, 1479, 1462, 1410, 1338, 1235, 1206, 1004, 748, 698 cm^{-1}.

(*R*)-1-((*R*)-2-azido-4-phenylbutanoyl)indoline-2-carboxylic acid (**7b**)

Synthesized from **6b** (691 mg, 1.9 mmol) using the general procedure described in **6.7.** White solid, yield: 631 mg (95%). 1H NMR (DMSO-d_6, 400 MHz): δ 2.07-2.13 (m, 1H, -CHCH$_2$CH$_2$Ph), 2.17-2.25 (m, 1H, -CHCH$_2$CH$_2$Ph), 2.68-2.83 (m, 2H, -CHCH$_2$CH$_2$Ph), 3.24-3.35 (m, 1H, -NHCHCH$_2$-), 3.62 (dd, 1H, *J* = 16.6 Hz, *J* = 10.8 Hz, -NHCHCH$_2$-), 3.95 (dd, 1H, *J* = 9.4 Hz, *J* = 4.6 Hz, -CHCH$_2$CH$_2$Ph), 5.04 (dd, 1H, *J* = 10.7 Hz, *J* = 1.8 Hz, -NHCHCH$_2$-), 7.08 (dt, 1H, *J* = 7.4 Hz, *J* = 0.9 Hz, indoline Ar-H), 7.20-7.30 (m, 7H, indoline Ar-H), 8.12 (d, 1H, *J* = 8.0 Hz, indoline Ar-H), 12.76 (bs, 1H, -COOH) ppm. HRMS for $C_{19}H_{17}N_4O_3$: calculated 335.1144; found 335.1141. IR(ATR): *ν* = 3028, 2927, 2105, 1733, 1662, 1599, 1481, 1461, 1410, 1339, 1239, 1208, 751, 699 cm^{-1}.

(*S*)-1-((*R*)-2-Azido-3-phenylpropanoyl)indoline-2-carboxylic acid (**7c**)

Synthesized from **6c** (665 mg, 1.9 mmol) using the general procedure described in **6.7.** White solid, yield: 600 mg (94%). ^{1}H NMR (MeOH-d_4, 400 MHz): δ 2.97 (dd, 1H, *J* = 16.3 Hz, *J* = 10.5 Hz, -CHCH$_2$Ph), 3.13-3.20 (m, 3H, -NHCHCH$_2$- + -CHCH$_2$Ph), 4.25 (t, 1H, *J* = 7.7 Hz, -CHCH$_2$Ph), 4.41 (dd, 1H, *J* = 10.5 Hz, *J* = 2.1 Hz, -NHCHCH$_2$-), 7.06 (dt, 1H, *J* = 7.4 Hz, *J* = 0.9 Hz, indoline Ar-H), 7.14 (d, 1H, *J* = 7.3 Hz, indoline Ar-H), 7.20-7.28 (m, 6H, -CHCH$_2$Ph + indoline Ar-H), 8.21 (d, 1H, *J* = 8.1 Hz, indoline Ar-H) ppm.^{13}C NMR (MeOH-d_4, 100 MHz): δ 173.97, 171.02, 142.86, 137.11, 131.07, 130.57, 129.87, 128.67, 128.47, 126.05, 125.62, 118.62, 63.24, 61.74, 39.68, 33.74 ppm. HRMS for $C_{18}H_{15}N_4O_3$: calculated 335.1144; found 335.1142. IR(ATR): *ν* = 3030, 2924, 2104, 1736, 1710, 1660, 1625, 1598, 1480, 1462, 1409, 1341, 1188, 1168, 1077, 883, 747, 700, 640 cm^{-1}.

(*S*)-1-((*R*)-2-Azido-4-phenylbutanoyl)indoline-2-carboxylic acid (**7d**)

Synthesized from **6d** (655 mg, 1.9 mmol) using the general procedure described in **6.7.** White solid, yield: 598 mg (95%). ^{1}H NMR (DMSO-d_6, 400 MHz): δ 1.97-2.06 (m, 1H, -CHCH$_2$CH$_2$Ph), 2.17-2.25 (m, 1H, -CHCH$_2$CH$_2$Ph), 2.68-2.83 (m, 2H, -CHCH$_2$CH$_2$Ph), 3.24-3.35 (m, 1H, -NHCHCH$_2$-), 3.60 (dd, 1H, *J* = 16.5 Hz, *J* = 10.8 Hz, -NHCHCH$_2$-), 3.94 (dd, 1H, *J* = 9.4 Hz, *J* = 4.6 Hz, -CHCH$_2$CH$_2$Ph), 5.04 (dd, 1H, *J* = 10.7 Hz, *J* = 1.8 Hz, -NHCHCH$_2$-), 7.08 (dt, 1H, *J* = 7.4 Hz, *J* = 0.9 Hz, indoline Ar-H), 7.20-7.30 (m, 7H, indoline Ar-H), 8.13 (d, 1H, *J* = 8.0 Hz, indoline Ar-H), 13.51 (bs, 1H, -COOH) ppm. HRMS for $C_{19}H_{17}N_4O_3$: calculated 349.1301; found 349.1306. IR(ATR): *ν* = 3027, 2930, 2106, 1733, 1664, 1598, 1480, 1463, 1410, 1338, 1236, 1207, 1004, 756, 698 cm^{-1}.

Methyl *N*^{2}-((*R*)-1-((*R*)-2-azido-4-phenylbutanoyl)indoline-2-carbonyl)-*N*$^\omega$-nitro-*L*-argininate (**8b**)

Synthesized from **7b** (280 mg, 0.8 mmol) using the general coupling procedure type A. White solid, yield: 375 mg (83%). ^{1}H NMR (DMSO-d_6, 400 MHz): δ 1.49-1.57 (m, 2H, -CHCH$_2$CH$_2$CH$_2$NH-), 1.62-1.67 (m, 1H, -CHCH$_2$CH$_2$CH$_2$NH-), 1.70-1.79 (m, 1H, -CHCH$_2$CH$_2$CH$_2$NH-), 2.05-2.10 (m, 2H, -CHCH$_2$CH$_2$Ph), 2.56-2.60 (m, 1H, -CHCH$_2$CH$_2$Ph), 2.66-2.69 (m, 1H, -CHCH$_2$CH$_2$Ph), 3.07-3.17 (m, 3H, -CH$_2$OH + -CHCH$_2$CH$_2$CH$_2$NH), 3.30-3.40 (m, 1H, -CH$_2$OH), 3.61-3.74 (m, 6H, -COOMe + -NHCHCH$_2$- + -CHCH$_2$CH$_2$CH$_2$NH), 4.10 (dd, 1H, *J* = 12.8 Hz, *J* = 6.6 Hz, -CHCH$_2$CH$_2$Ph), 5.18 (d, 1H, *J* = 10.2 Hz, -NHCHCH$_2$-), 7.07 (t, 1H, *J* = 7.4 Hz, indoline Ar-H), 7.19-7.33 (m, 7H, indoline Ar-H + -CHCH$_2$CH$_2$Ph), 8.11 (d, 1H, *J* = 7.9 Hz, indoline Ar-H), 8.58 (bs, 1H, guanidine-NH), 8.78 (d, 1H, *J* = 6.4 Hz, -CONH-), ppm. HRMS for $C_{26}H_{32}N_9O_6$: calculated 566.2476; found 566.2472. IR(ATR): *ν* = 3297, 2952, 2103, 1742, 1656, 1597, 1537, 1482, 1409, 1254, 1210, 1153, 1097, 1002, 752, 700 cm^{-1}.

(*R*)-1-((*R*)-2-Azido-3-phenylpropanoyl)-*N*-((*S*)-1-hydroxy-5-(3-nitroguanidino)pentan-2-yl)indoline-2-carboxamide (**8c**)

Synthesized from **7a** (302 mg, 0.9 mmol) using the general coupling procedure type B. White solid, yield: 259 mg (55%). ^{1}H NMR (DMSO-d_6, 400 MHz): δ 1.41-1.62 (m, 4H, -CHCH$_2$CH$_2$CH$_2$NH-), 3.05-3.17 (m, 5H, -CHCH$_2$CH$_2$CH$_2$NH- + -CHCH$_2$Ph + -NHCHCH$_2$-), 3.30-3.38 (m, 1H, -CH$_2$OH), 3.65-3.72 (m, 2H, -NHCHCH$_2$- + -CH$_2$OH), 3.86 (dd, 1H, *J* = 10.0 Hz, *J* = 3.9 Hz, -CHCH$_2$CH$_2$CH$_2$NH-), 4.78 (t, 1H, *J* = 5.4 Hz, -CHCH$_2$Ph), 5.25 (dd, 1H, *J* = 11.0 Hz, *J* = 2.6 Hz, -NHCHCH$_2$-), 7.08 (t, 1H, *J* = 7.3 Hz, indoline Ar-H), 7.22-7.36 (m, 7H, indoline Ar-H + -CHCH$_2$Ph), 8.15 (d, 1H, *J* = 8.1 Hz, indoline Ar-H), 8.19 (d, 1H, *J* = 8.2 Hz, CONH-), 8.53 (bs, 1H, guanidine-NH) ppm. IR(ATR): *ν* = 3640, 3298, 2951, 2870, 2108, 1639, 1596, 1540, 1482, 1412, 1262, 1214, 1150, 1080, 935, 862, 753, 697 cm^{-1}.

(*R*)-1-((*R*)-2-Azido-4-phenylbutanoyl)-*N*-((*S*)-1-hydroxy-5-(3-nitroguanidino)pentan-2-yl)indoline-2-carboxamide (**8d**)

Synthesized from **7b** (300 mg, 0.86 mmol) using the general coupling procedure type B. White solid, yield: 240 mg (52%). ^{1}H NMR (DMSO-*d*$_6$, 400 MHz): δ 1.33-1.59 (m, 4H, -$CH\underline{CH_2CH_2}CH_2NH$-), 2.02-2.17 (m, 2H, -$CH\underline{CH_2}CH_2Ph$), 2.55-2.59 (m, 1H, -$CHCH_2\underline{CH_2}Ph$), 2.63-2.69 (m, 1H, -$CHCH_2\underline{CH_2}Ph$), 3.08-3.12 (m, 3H, -$CHCH_2CH_2\underline{CH_2}NH$- + -$NHCH\underline{CH_2}$-), 3.32-3.38 (m, 1H, -$\underline{CH_2}OH$), 3.64-3.73 (m, 3H, -$\underline{CH_2}OH$ + -$NHCH\underline{CH_2}$- + -$\underline{CH}CH_2CH_2CH_2NH$-), 4.77 (t, 1H, *J* = 5.5 Hz, -$\underline{CH}CH_2CH_2Ph$), 5.15 (dd, 1H, *J* = 10.7 Hz, *J* = 2.3 Hz, -$NH\underline{CH}CH_2$-), 7.07 (t, 1H, *J* = 7.4 Hz, indoline Ar-$\underline{H}$), 7.19-7.32 (m, 7H, indoline Ar-$\underline{H}$ + -$CHCH_2CH_2\underline{Ph}$), 8.13 (d, 1H, *J* = 8.1 Hz, indoline Ar-$\underline{H}$), 8.16 (d, 1H, *J* = 8.4 Hz, -$CO\underline{NH}$-), 8.56 (bs, 1H, guanidine-$\underline{NH}$) ppm. HRMS for $C_{25}H_{30}N_9O_5$: calculated 536.2370; found 536.2383. IR(ATR): ν = 3304, 2937, 2102, 1628, 1595, 1538, 1482, 1407, 1528, 1152, 1081, 1026, 750, 699 cm^{-1}.

Methyl *N*^{2}-((*S*)-1-((*R*)-2-azido-3-phenylpropanoyl)indoline-2-carbonyl)-*N*$^\omega$-nitro-*L*-argininate (**8e**)

Synthesized from **7c** (269 mg, 0.8 mmol) using the general coupling procedure type A. White solid, yield: 375 mg (85%). ^{1}H NMR (DMSO-*d*$_6$, 400 MHz): δ 1.53-1.83 (m, 4H, -$CH\underline{CH_2CH_2}CH_2NH$-), 2.97 (dd, 1H, *J* = 14.2 Hz, *J* = 10.1 Hz, -$CH\underline{CH_2}Ph$), 3.07 (dd, 1H, *J* = 17.0 Hz, *J* = 2.7 Hz, -$CH\underline{CH_2}Ph$), 3.15-3.20 (m, 2H, -$CHCH_2CH_2\underline{CH_2}NH$-), 3.43 (m, 4H, -$COO\underline{Me}$ + -$NHCH\underline{CH_2}$-), 3.59 (dd, 1H, *J* = 16.8 Hz, *J* = 11.1 Hz, -$NHCH\underline{CH_2}$-), 3.90 (dd, 1H, *J* = 10.0 Hz, *J* = 4.4 Hz, -$\underline{CH}CH_2CH_2CH_2NH$-), 4.20-4.25 (m, 1H, -$\underline{CH}CH_2Ph$), 5.01 (dd, 1H, *J* = 11.0 Hz, *J* = 2.9 Hz, -$NH\underline{CH}CH_2$-), 7.07 (dt, 1H, *J* = 7.6 Hz, *J* = 1.0 Hz, indoline Ar-$\underline{H}$), 7.21-7.38 (m, 7H, indoline Ar-$\underline{H}$ + -$CHCH_2\underline{Ph}$), 8.16 (d, 1H, *J* = 7.8 Hz, indoline Ar-$\underline{H}$), 8.56 (bs, 1H, guanidine-$\underline{NH}$), 8.93 (d, 1H, J = 7.2 Hz, -$CO\underline{NH}$-) ppm. HRMS for $C_{25}H_{28}N_9O_6$: calculated 550.2163; found 550.2160. IR(ATR): ν = 3295, 2949, 2107, 1739, 1655, 1626, 1597, 1535, 1481, 1406, 1254, 1151, 1080, 1020, 753, 699 cm^{-1}.

Methyl *N*^{2}-((*S*)-1-((*R*)-2-azido-4-phenylbutanoyl)indoline-2-carbonyl)-*N*$^\omega$-nitro-*L*-argininate (**8f**)

Synthesized from **7d** (270 mg, 0.77 mmol) using the general coupling procedure type A. White solid, yield: 374 mg (86%). ^{1}H NMR (DMSO-*d*$_6$, 400 MHz): δ 1.55-1.58 (m, 2H, -$CHCH_2\underline{CH_2}CH_2NH$-), 1.63-1.72 (m, 1H, -$CH\underline{CH_2}CH_2CH_2NH$-), 1.77-1.84 (m, 1H, -$CH\underline{CH_2}CH_2CH_2NH$-), 1.93-2.00 (m, 1H, -$CH\underline{CH_2}CH_2Ph$), 2.24-2.31 (m, 1H, -$CH\underline{CH_2}CH_2Ph$), 2.68-2.79 (m, 2H, -$CHCH_2\underline{CH_2}Ph$), 3.06-3.19 (m, 3H, -$NHCH\underline{CH_2}$- + -$CHCH_2CH_2\underline{CH_2}NH$), 3.65-3.74 (m, 4H, -$COO\underline{Me}$ + - $NHCH\underline{CH_2}$-), 3.82 (dd, 1H, *J* = 9.7 Hz, *J* = 3.9 Hz, -$\underline{CH}CH_2CH_2CH_2NH$-), 4.23 (dd, 1H, *J* = 13.2 Hz, *J* =7.9 Hz, -$\underline{CH}CH_2CH_2Ph$), 5.06 (dd, 1H, *J* = 11.0 Hz, *J* = 3.0 Hz, -$NH\underline{CH}CH_2$-), 7.06 (dt, 1H, *J* = 7.5 Hz, *J* = 0.8 Hz, indoline Ar-$\underline{H}$), 7.19-7.33 (m, 7H, indoline Ar-$\underline{H}$ + -$CHCH_2CH_2\underline{Ph}$), 8.14 (d, 1H, *J* = 8.0 Hz, indoline Ar-$\underline{H}$), 8.55 (bs, 1H, guanidine-$\underline{NH}$), 8.86 (d, 1H, *J* = 7.3 Hz, -$CO\underline{NH}$-), ppm. HRMS for $C_{26}H_{32}N_9O_6$: calculated 566.2476; found 566.2472. IR(ATR): ν = 3296, 3063, 2931, 2104, 1742, 1656, 1598, 1537, 1482, 1408, 1388, 1255, 1153, 1096, 1021, 756, 701, 661 cm^{-1}.

(*S*)-1-((*R*)-2-Azido-3-phenylpropanoyl)-*N*-((*S*)-1-hydroxy-5-(3-nitroguanidino)pentan-2-yl)indoline-2-carboxamide (**8g**)

Synthesized from **7c** (302 mg, 0.9 mmol) using the general coupling procedure type B. White solid, yield: 264 mg (56%). ^{1}H NMR (DMSO-*d*$_6$, 400 MHz): δ 1.41-1.64 (m, 4H, -$CH\underline{CH_2CH_2}CH_2NH$-), 2.99-3.05 (m, 2H, -$CHCH_2CH_2\underline{CH_2}NH$-), 3.11-3.16 (m, 2H, -$CH\underline{CH_2}Ph$), 3.22-3.27 (m, 2H, -$NHCH\underline{CH_2}$- + -$\underline{CH_2}OH$), 3.48 (dd, 1H, *J* = 16.9 Hz, *J* = 11.0 Hz, -$\underline{CH_2}OH$), 3.65-3.72 (m, 1H, -$NHCH\underline{CH_2}$-), 3.87 (dd, 1H, *J* = 9.3 Hz, *J* = 5.3 Hz, -$\underline{CH}CH_2CH_2CH_2NH$-), 4.77 (t, 1H, *J* = 5.4 Hz, -$\underline{CH}CH_2Ph$), 4.88 (dd, 1H, *J* = 10.9 Hz, *J* = 3.3 Hz, -$NH\underline{CH}CH_2$-), 7.06 (dt, 1H, *J* = 7.5 Hz, *J* = 1.0 Hz, indoline Ar-$\underline{H}$), 7.20-7.33 (m, 7H, indoline Ar-$\underline{H}$ + -$CHCH_2\underline{Ph}$), 8.16 (d, 1H, *J* = 7.9 Hz, indoline Ar-$\underline{H}$), 8.27 (d, 1H, *J* = 8.5 Hz, -$CO\underline{NH}$-), 8.53 (bs, 1H, guanidine-$\underline{NH}$) ppm. IR(ATR): ν = 3639, 3297, 2954, 2873, 2104, 1636, 1598, 1540, 1482, 1413, 1263, 1214, 1150, 1081, 934, 866, 750, 697 cm^{-1}.

(*S*)-1-((*R*)-2-Azido-4-phenylbutanoyl)-*N*-((*S*)-1-hydroxy-5-(3-nitroguanidino)pentan-2-yl)indoline-2-carboxamide (**8h**)

Synthesized from **7d** (280 mg, 0.8 mmol) using the general coupling procedure type B. White solid, yield: 219 mg (51%). ^{1}H NMR (DMSO-*d*$_6$, 400 MHz): δ 1.32-1.63 (m, 4H, -$CH\underline{CH_2CH_2}CH_2NH$-), 1.94-2.04 (m, 1H, -$CH\underline{CH_2}CH_2Ph$), 2.20-2.29 (m, 1H, -$CH\underline{CH_2}CH_2Ph$), 2.67-2.73 (m, 1H, -$CHCH_2\underline{CH_2}Ph$), 2.76-2.83

(m, 1H, $-CHCH_2\underline{CH_2}Ph$), 3.05 (dd, 1H, *J* = 16.7, *J* = 2.6 Hz, $-NHCH\underline{CH_2}-$), 3.12-3.17 (m, 2H, $-CHCH_2CH_2\underline{CH_2}NH-$), 3.23-3.29 (m, 1H, $-\underline{CH_2}OH$), 3.63-3.74 (m, 3H, $-\underline{CH_2}OH$ + $-NHCH\underline{CH_2}-$ + $-\underline{CH}CH_2CH_2CH_2NH-$), 4.78 (t, 1H, *J* = 5.4 Hz, $-\underline{CH}CH_2CH_2Ph$), 4.94 (dd, 1H, *J* = 10.9 Hz, *J* = 3.1 Hz, $-NH\underline{CH}CH_2-$), 7.05 (dt, 1H, *J* = 7.5 Hz, *J* = 0.8 Hz, indoline Ar-$\underline{H}$), 7.19-7.32 (m, 7H, indoline Ar-$\underline{H}$ + $-CHCH_2CH_2\underline{Ph}$), 8.12-8.15 (m, 2H, indoline Ar-$\underline{H}$ + $-CO\underline{NH}-$), 8.54 (bs, 1H, guanidine-$\underline{NH}$) ppm. ^{13}C NMR (DMSO-d_6, 100 MHz): δ 170.43, 168.79, 159.26, 142.87, 140.39, 130.23, 128.67, 128.55, 128.42, 128.16, 127.16, 126.11, 124.54, 124.00, 116.36, 63.03, 60.34, 59.95, 54.90, 50.69, 40.52, 34.42, 32.47, 31.56, 27.87, 24.84 ppm. HRMS for $C_{25}H_{30}N_9O_5$: calculated 536.2370; found 536.2383. IR(ATR): ν = 3296, 2931, 2864, 2103, 1629, 1596, 1541, 1481, 1407, 1346, 1259, 1153, 1047, 1023, 1002, 822, 755, 700 cm^{-1}.

Methyl ^{N}2-((*R*)-1-((*R*)-2-((*R*)-2-hydroxy-3-phenylpropanamido)-4-phenylbutanoyl)indoline-2-carbonyl)-*N*$^{\omega}$-nitro-*L*-argininate (**10b**)

Synthesized from **9b** (110 mg, 0.205 mmol) using the general coupling procedure type C. White solid, yield: 83 mg (59%). ^{1}H NMR (DMSO-d_6, 400 MHz): δ 1.49-1.79 (m, 4H, $-CH\underline{CH_2CH_2}CH_2NH-$), 1.84-1.94 (m, 1H, $-CH(OH)\underline{CH_2}Ph$), 2.27-2.32 (m, 1H, $-CH(OH)\underline{CH_2}Ph$), 2.47-2.53 (m, 1H, $-CH\underline{CH_2}CH_2Ph$), 2.78 (dd, 1H, *J* = 13.7, *J* = 7.8 Hz, $-CH\underline{CH_2}CH_2Ph$), 2.99 (dd, 1H, *J* = 13.4 Hz, *J* = 4.0 Hz, $-CHCH_2\underline{CH_2}Ph$), 3.09-3.21 (m, 3H, $-CHCH_2CH_2\underline{CH_2}NH-$ + $-CHCH_2\underline{CH_2}Ph$), 3.60-3.63 (m, 4H, $-COO\underline{Me}$ + $-NHCH\underline{CH_2}-$), 4.06-4.11 (m, 1H, $-NHCH\underline{CH_2}-$), 4.16-4.20 (m, 1H, $-\underline{CH}CH_2CH_2CH_2NH-$), 4.26-4.33 (m, 1H, $-\underline{CH}CH_2CH_2Ph$), 5.54 (dd, 1H, *J* = 11.3 Hz, *J* = 3.0 Hz, $-NH\underline{CH}CH_2-$), 5.60 (d, 1H, *J* = 6.0 Hz, $-\underline{CH}(OH)CH_2Ph$), 7.03 (dt, 1H, *J* = 7.3 Hz, *J* = 0.7 Hz, indoline Ar-$\underline{H}$), 7.11-7.30 (m, 12H, indoline Ar-$\underline{H}$ + -CHCH2CH2$\underline{Ph}$ + $-CH(OH)CH_2\underline{Ph}$), 8.09 (d, 1H, *J* = 8.0 Hz, indoline Ar-$\underline{H}$), 8.12 (d, 1H, *J* = 8.1 Hz, $-CO\underline{NH}-$), 8.58 (bs, 1H, guanidine-$\underline{NH}$), 8.85 (d, 1H, *J* = 6.9 Hz, $-CO\underline{NH}-$) ppm. HRMS for $C_{35}H_{40}N_7O_8$: calculated 686.2938; found 686.2954. IR(ATR): ν = 3292, 3028, 2951, 1740, 1630, 1596, 1528, 1482, 1413, 1347, 1256, 1151, 1087, 1023, 1002, 750, 699, 621 cm^{-1}.

(*R*)-1-((*R*)-2-((*R*)-2-Hydroxy-3-phenylpropanamido)-3-phenylpropanoyl)-*N*-((*S*)-1-hydroxy-5-(3-nitroguanidino)pentan-2-yl)indoline-2-carboxamide (**10c**)

Synthesized from **9c** (189 mg, 0.38 mmol) using the general coupling procedure type C. White solid, yield: 127 mg (52%). ^{1}H NMR (DMSO-d_6, 400 MHz): δ 1.36-1.66 (m, 4H, $-CH\underline{CH_2CH_2}CH_2NH-$), 2.74-2.80 (m, 2H, $-CH(OH)\underline{CH_2}Ph$), 2.99-3.02 (m, 2H, $-CH\underline{CH_2}Ph$), 3.10-3.17 (m, 3H, $-CHCH_2CH_2\underline{CH_2}NH-$ + $-NHCH\underline{CH_2}$), 3.60 (dd, 1H, *J* = 16.9 Hz, *J* = 10.9 Hz, $-\underline{CH_2}OH$), 3.71-3.75 (m, 1H, $-\underline{CH_2}OH$), 3.99-4.04 (m, 1H, $-NHCH\underline{CH_2}$), 4.47-4.52 (m, 1H, $-\underline{CH}CH_2CH_2CH_2NH-$), 4.75 (t, 1H, *J* = 5.4 Hz, $-\underline{CH}CH_2Ph$), 5.44 (d, 1H, *J* = 6.1 Hz, $-NH\underline{CH}CH_2-$), 5.53 (dd, 1H, *J* = 11.5 Hz, *J* = 3.0 Hz, $-\underline{CH}(OH)CH_2Ph$), 7.03-7.28 (m, 13H, indoline Ar-$\underline{H}$ + $-CHCH_2\underline{Ph}$ + $-CH(OH)CH_2\underline{Ph}$), 8.14 (d, 1H, *J* = 7.7 Hz, $-CO\underline{NH}-$), 8.15 (d, 1H, *J* = 7.9 Hz, indoline Ar-$\underline{H}$), 8.20 (d, 1H, *J* = 8.2 Hz, $-CO\underline{NH}-$), 8.52 (bs, 1H, guanidine-$\underline{NH}$) ppm. IR(ATR): ν = 3277, 2940, 1628, 1597, 1530, 1481, 1410, 1261, 1177, 1049, 1019, 953, 882, 748, 698, 629 cm^{-1}.

(*R*)-1-((*R*)-2-((*R*)-2-Hydroxy-3-phenylpropanamido)-4-phenylbutanoyl)-*N*-((*S*)-1-hydroxy-5-(3-nitroguanidino)pentan-2-yl)indoline-2-carboxamide (**10d**)

Synthesized from **9d** (82 mg, 0.16 mmol) using the general coupling procedure type C. White solid, yield: 56 mg (53%). ^{1}H NMR (DMSO-d_6, 400 MHz): δ 1.46-1.55 (m, 2H, $-CHCH_2\underline{CH_2}CH_2NH-$), 1.68-1.83 (m, 2H, $-CH\underline{CH_2}CH_2CH_2NH-$), 1.88-1.95 (m, 1H, $-CH(OH)\underline{CH_2}Ph$), 2.07-2.12 (m, 1H, $-CH(OH)\underline{CH_2}Ph$), 2.46-2.50 (m, 1H, $-CH\underline{CH_2}CH_2Ph$), 2.80 (dd, 1H, *J* = 13.7 Hz, *J* = 8.0 Hz, $-CH\underline{CH_2}CH_2Ph$), 3.00-3.06 (m, 2H, $-CHCH_2\underline{CH_2}Ph$), 3.01-3.19 (m, 2H, $-CHCH_2CH_2\underline{CH_2}NH-$), 3.30-3.43 (m, 1H, $-\underline{CH_2}OH$), 3.61-3.69 (m, 2H, $-\underline{CH_2}OH$ + $-NHCH\underline{CH_2}-$), 4.14-4.19 (m, 1H, $-NHCH\underline{CH_2}-$), 4.22-4.27 (m, 1H, $-\underline{CH}CH_2CH_2CH_2NH-$), 4.45-4.50 (m, 1H, $-\underline{CH}CH_2CH_2Ph$), 5.10 (d, 1H, *J* = 11.0 Hz, $-NH\underline{CH}CH_2-$), 5.95 (d, 1H, *J* = 5.8 Hz, $-\underline{CH}(OH)CH_2Ph$), 7.04 (t, 1H, *J* = 7.5 Hz, indoline Ar-$\underline{H}$), 7.16-7.33 (m, 12H, indoline Ar-$\underline{H}$ + -CHCH2CH2$\underline{Ph}$ + $-CH(OH)CH_2\underline{Ph}$), 7.82 (d, 1H, *J* = 7.6 Hz, $-CO\underline{NH}-$), 8.10 (d, 1H, *J* = 8.0 Hz, indoline Ar-$\underline{H}$), 8.57 (bs, 1H, guanidine-$\underline{NH}$), 8.80 (d, 1H, *J* = 7.6 Hz, $-CO\underline{NH}-$) ppm. HRMS for $C_{34}H_{40}N_7O_7$: calculated 658.2989; found 658.2993. IR(ATR): ν = 3288, 2099, 1625, 1554, 1529, 1481, 1406, 1255, 1152, 1085, 1026, 750, 698, 605 cm^{-1}.

Methyl *N*^{2}-((*R*)-1-((*R*)-2-((*R,S*)-2-hydroxy-3-(4-hydroxyphenyl)propanamido)-4-phenylbutanoyl)indoline-2-carbonyl)-*N*$^{\omega}$-nitro-*L*-argininate (**10e**)

Synthesized from **9b** (110 mg, 0.205 mmol) using the general coupling procedure type C. White solid, yield: 74 mg (52%). ^{1}H NMR (DMSO-d_6, 400 MHz): δ 1.52-1.79 (m, 4H, -CHCH$_2$CH$_2$CH$_2$NH-), 1.85-1.96 (m, 2H, -CH(OH)CH$_2$Ph(OH)), 2.30-2.38 (m, 1H, -CHCH$_2$CH$_2$Ph), 2.63-2.68 (m, 1H, -CHCH$_2$CH$_2$Ph), 2.82-2.90 (m, 1H, -CHCH$_2$CH$_2$Ph), 3.09-3.21 (m, 3H, -CHCH$_2$CH$_2$CH$_2$NH- + -CHCH$_2$CH$_2$Ph), 3.55-3.66 (m, 4H, -COOMe + -NHCHCH$_2$-), 4.06-4.13 (m, 2H, -NHCHCH$_2$- + -CHCH$_2$CH$_2$CH$_2$NH-), 4.24-4.33 (m, 1H, -CHCH$_2$CH$_2$Ph), 5.38 (d, 1/2H, *J* = 6.3 Hz, -NHCHCH$_2$-), 5.49-5.57 (m, 3/2H, -NHCHCH$_2$- + CH(OH)CH$_2$Ph(OH)), 6.58-6.61 (m, 2H, -CH(OH)CH$_2$Ph(OH)), 6.98-7.30 (m, 10H, indoline Ar-H + -CHCH$_2$CH$_2$Ph + -CH(OH)CH$_2$Ph(OH)), 8.08-8.11 (m, 2H, indoline Ar-H + -CONH-), 8.57 (bs, 1H, guanidine-NH), 8.83-8.84 (m, 1H, -CONH-), 9.10(9.12) (s, 1H, -CH(OH)CH$_2$Ph(OH)) ppm. HRMS for $C_{35}H_{40}N_7O_9$: calculated 702.2888; found 702.2896. IR(ATR): ν = 3297, 2949, 1738, 1631, 1595, 1514, 1482, 1413, 1347, 1259, 1105, 1020, 826, 752, 699 cm^{-1}.

(*R*)-1-((*R*)-2-((*R,S*)-2-Hydroxy-3-(4-hydroxyphenyl)propanamido)-4-phenylbutanoyl)-*N*-((*S*)-1-hydroxy-5-(3-nitroguanidino)pentan-2-yl)indoline-2-carboxamide (**10f**)

Synthesized from **9d** (77 mg, 0.15 mmol) using the general coupling procedure type C. White solid, yield: 50 mg (50%). ^{1}H NMR (DMSO-d_6, 400 MHz): δ 1.34-1.51 (m, 2H, -CHCH$_2$CH$_2$CH$_2$NH-), 1.61-1.64 (m, 2H, -CHCH$_2$CH$_2$CH$_2$NH-), 1.89-2.00 (m, 2H, -CH(OH)CH$_2$Ph(OH)), 2.31-2.39 (m, 1H, -CHCH$_2$CH$_2$Ph), 2.61-2.68 (m, 1H, -CHCH$_2$CH$_2$Ph), 2.83-2.90 (m, 1H, -CHCH$_2$CH$_2$Ph), 3.08-3.19 (m, 3H, -CHCH$_2$CH$_2$CH$_2$NH- + -CHCH$_2$CH$_2$Ph), 3.30-3.36 (m, 1H, -CH$_2$OH), 3.56-3.69 (m, 2H -CH$_2$OH + -NHCHCH$_2$-), 4.06-4.13 (m, 1H, -NHCHCH$_2$-), 4.34-4.41 (m, 1H, -CHCH$_2$CH$_2$CH$_2$NH-), 4.73-4.76 (m, 1H, -CHCH$_2$CH$_2$Ph), 5.38-5.48 (m, 2H, -NHCHCH$_2$- + -CH(OH)CH$_2$Ph(OH)), 6.58-6.61 (m, 2H, -CH(OH)CH$_2$Ph(OH), 6.98-7.30 (m, 10H, indoline Ar-H + -CHCH$_2$CH$_2$Ph + -CH(OH)CH$_2$Ph(OH)), 8.07-8.21 (m, 3H, indoline Ar-H + -CONH-), 8.54 (bs, 1H, guanidine-NH), 9.10(9.11) (s, 1H, -CH(OH)CH$_2$Ph(OH)) ppm. HRMS for $C_{34}H_{40}N_7O_8$: calculated 674.2938; found 674.2945. IR(ATR): ν = 3299, 2928, 1630, 1594, 1514, 1482, 1412, 1345, 1259, 1105, 1081, 1023, 751, 699 cm^{-1}.

Methyl N^2-((*S*)-1-(((*R*)-2-hydroxy-3-phenylpropanoyl)-*D*-phenylalanyl)indoline-2-carbonyl)-N^ω-nitro-*L*-argininate (**10g**)

Synthesized from **9e** (120 mg, 0.23 mmol) using the general coupling procedure type C. White solid, yield: 92 mg (60%). ^{1}H NMR (DMSO-d_6, 400 MHz): δ 1.46-1.79 (m, 4H, -CHCH$_2$CH$_2$CH$_2$NH-), 2.64 (dd, 1H, *J* = 13.7 Hz, *J* = 8.3 Hz, -CH(OH)CH$_2$Ph), 2.92-3.05 (m, 4H, -CH(OH)CH$_2$Ph + -CHCH$_2$Ph + -NHCHCH$_2$-), 3.12-3.18 (m, 2H, -CHCH$_2$CH$_2$CH$_2$NH-), 3.61 (s, 3H, -COOMe), 4.05-4.09 (m, 1H, -NHCHCH$_2$-), 4.21 (dd, 1H, *J* = 13.0 Hz, *J* = 8.0 Hz, -CHCH$_2$CH$_2$CH$_2$NH-), 4.63 (dd, 1H, *J* = 13.6 Hz, *J* = 6.2 Hz, -CHCH$_2$Ph), 4.90 (dd, 1H, *J* = 10.8 Hz, *J* = 2.5 Hz, -NHCHCH$_2$-), 5.89 (d, 1H, *J* = 5.7 Hz, -CH(OH)CH$_2$Ph), 7.03-7.05 (m, 3H, indoline Ar-H + -CHCH$_2$Ph), 7.20-7.26 (m, 10H, indoline Ar-H + -CHCH$_2$Ph + -CH(OH)CH$_2$Ph), 7.67 (d, 1H, *J* = 8.0 Hz, -CONH-), 8.04 (d, 1H, *J* = 8.0 Hz, indoline Ar-H), 8.53 (bs, 1H, guanidine-NH), 8.82 (d, 1H, *J* = 7.6 Hz, -CONH-) ppm. HRMS for $C_{34}H_{38}N_7O_8$: calculated 672.2782; found 672.2793. IR(ATR): ν = 3293, 2930, 1741, 1652, 1598, 1528, 1481, 1409, 1254, 1089, 1026, 754, 700, 661 cm^{-1}.

Methyl N^2-((*R*)-1-((*R*)-2-((*R*)-2-hydroxy-3-phenylpropanamido)-4-phenylbutanoyl)indoline-2-carbonyl)-N^ω-nitro-*L*-argininate (**10h**)

Synthesized from **9f** (100 mg, 0.186 mmol) using the general coupling procedure type C. White solid, yield: 78 mg (61%). ^{1}H NMR (DMSO-d_6, 400 MHz): δ 1.46-1.53 (m, 2H, -CHCH$_2$CH$_2$CH$_2$NH-), 1.68-1.85 (m, 2H, -CHCH$_2$CH$_2$CH$_2$NH-), 2.06-2.11 (m, 1H, -CH(OH)CH$_2$Ph), 2.27-2.32 (m, 1H, -CH(OH)CH$_2$Ph), 2.45-2.53 (m, 1H, -CHCH$_2$CH$_2$Ph), 2.81 (dd, 1H, *J* = 13.8 Hz, *J* = 7.8 Hz, -CHCH$_2$CH$_2$Ph), 3.03 (dd, 1H, *J* = 13.8 Hz, *J* = 2.9 Hz, -CHCH$_2$CH$_2$Ph), 3.11-3.18 (m, 3H, -CHCH$_2$CH$_2$CH$_2$NH- + -CHCH$_2$CH$_2$Ph), 3.62-3.67 (m, 4H, -COOMe + -NHCHCH$_2$-), 4.15-4.19 (m, 1H, -NHCHCH$_2$-), 4.22-4.27 (m, 1H, -CHCH$_2$CH$_2$CH$_2$NH-), 4.45-4.50 (m, 1H, -CHCH$_2$CH$_2$Ph), 5.11 (dd, 1H, *J* = 10.7 Hz, *J* = 2.2 Hz, -NHCHCH$_2$-), 5.96 (d, 1H, *J* = 5.7 Hz, -CH(OH)CH$_2$Ph), 7.04 (t, 1H, *J* = 7.4 Hz, indoline Ar-H), 7.16-7.32 (m, 12H, indoline Ar-H + -CHCH$_2$CH2Ph + -CH(OH)CH$_2$Ph), 7.82 (d, 1H, *J* = 7.5 Hz, -CONH-), 8.10 (d, 1H, *J* = 8.1 Hz, indoline Ar-H), 8.58 (bs, 1H, guanidine-NH), 8.80 (d, 1H, *J* = 7.7 Hz, -CONH-) ppm. HRMS

for $C_{35}H_{40}N_7O_8$: calculated 686.2938; found 686.2954. IR(ATR): ν = 3311, 2951, 1739, 1627, 1528, 1481, 1408, 1259, 1087, 1023, 755, 698, 619 cm^{-1}.

(*S*)-1-((*R*)-2-((*R*)-2-Hydroxy-3-phenylpropanamido)-3-phenylpropanoyl)-*N*-((*S*)-1-hydroxy-5-(3-nitroguanidino)pentan-2-yl)indoline-2-carboxamide (**10i**)

Synthesized from **9g** (104 mg, 0.20 mmol) using the general coupling procedure type C. White solid, yield: 69 mg (54%). ^{1}H NMR (DMSO-d_6, 400 MHz): δ 1.36-1.66 (m, 4H, $-CH\underline{CH_2CH_2}CH_2NH-$), 2.61-2.68 (m, 1H, $-CH(OH)\underline{CH_2}Ph$), 2.84-3.01 (m, 4H, $-CH(OH)\underline{CH_2}Ph$ + $-CH\underline{CH_2}Ph$ + $-NHCH\underline{CH_2}$), 3.13-3.17 (m, 2H, $-CHCH_2CH_2\underline{CH_2}NH-$), 3.44-3.45 (m, 1H, $-\underline{CH_2}OH$), 3.62-3.64 (m, 1H, $-\underline{CH_2}OH$), 4.04-4.06 (m, 1H, $-NHCH\underline{CH_2}$), 4.67-4.72 (m, 2H, $-\underline{CH}CH_2CH_2CH_2NH-$ + $-\underline{CH}CH_2Ph$), 4.90 (dd, 1H, *J* = 10.8 Hz, *J* = 2.7 Hz, $-NH\underline{CH}CH_2-$), 5.91 (d, 1H, *J* = 5.3 Hz, $-\underline{CH}(OH)CH_2Ph$), 7.00-7.05 (m, 3H, indoline Ar-$\underline{H}$ + $-CHCH_2\underline{Ph}$), 7.16-7.30 (m, 10H, indoline Ar-$\underline{H}$ + $-CHCH_2\underline{Ph}$ + $-CH(OH)CH_2\underline{Ph}$), 7.65 (d, 1H, *J* = 8.4 Hz, -CO$\underline{NH}$-), 8.04 (d, 1H, *J* = 7.9 Hz, indoline Ar-$\underline{H}$), 8.26 (d, 1H, *J* = 8.3 Hz,-CO$\underline{NH}$-), 8.54 (bs, 1H, guanidine-$\underline{NH}$) ppm. IR(ATR): ν = 3312, 2925, 1633, 1598, 1525, 1481, 1410, 1260, 1153, 1083, 1028, 749, 699, 617 cm^{-1}.

(*S*)-1-((*R*)-2-((*R*)-2-Hydroxy-3-phenylpropanamido)-4-phenylbutanoyl)-*N*-((*S*)-1-hydroxy-5-(3-nitroguanidino)pentan-2-yl)indoline-2-carboxamide (**10j**)

Synthesized from **9h** (77 mg, 0.15 mmol) using the general coupling procedure type C. White solid, yield: 49 mg (50%). ^{1}H NMR (DMSO-d_6, 400 MHz): δ 1.39-1.44 (m, 2H, $-CHCH_2\underline{CH_2}CH_2NH-$), 1.53-1.57 (m, 1H, $-CH\underline{CH_2}CH_2CH_2NH-$), 1.63-1.69 (m, 1H, $-CH\underline{CH_2}CH_2CH_2NH-$), 1.76-1.81 (m, 1H, $-CH(OH)\underline{CH_2}Ph$), 2.00-2.06 (m, 1H, $-CH(OH)\underline{CH_2}Ph$), 2.46-2.50 (m, 1H, $-CH\underline{CH_2}CH_2Ph$), 2.81 (dd, 1H, *J* = 13.8 Hz, *J* = 7.9 Hz, $-CH\underline{CH_2}CH_2Ph$), 3.00-3.05 (m, 2H, $-CHCH_2\underline{CH_2}Ph$), 3.12-3.18 (m, 2H, $-CHCH_2CH_2\underline{CH_2}NH-$), 3.49-3.53 (m, 1H, $-\underline{CH_2}OH$), 3.61-3.70 (m, 2H, $-\underline{CH_2}OH$ + $-NHCH\underline{CH_2}-$), 4.15-4.19 (m, 1H, $-NHCH\underline{CH_2}-$), 4.52-4.57 (m, 1H, $-\underline{CH}CH_2CH_2CH_2NH-$), 4.71 (t, 1H, *J* = 5.5 Hz, $-\underline{CH}CH_2CH_2Ph$), 5.04 (dd, 1H, *J* = 11.0 Hz, *J* = 2.5 Hz, $-NH\underline{CH}CH_2-$), 6.0 (d, 1H, *J* = 5.6 Hz, $-\underline{CH}(OH)CH_2Ph$), 7.03 (t, 1H, *J* = 7.4 Hz, indoline Ar-$\underline{H}$), 7.15-7.32 (m, 12H, indoline Ar-$\underline{H}$ + $-CHCH_2CH2\underline{Ph}$ + $-CH(OH)CH_2\underline{Ph}$), 7.80 (d, 1H, *J* = 8.1 Hz, -CO$\underline{NH}$-), 8.10 (d, 1H, *J* = 8.0 Hz, indoline Ar-$\underline{H}$), 8.22 (d, 1H, *J* = 8.5 Hz, -CO$\underline{NH}$-), 8.56 (bs, 1H, guanidine-$\underline{NH}$) ppm. HRMS for $C_{34}H_{40}N_7O_7$: calculated 658.2989; found 658.2993. IR(ATR): ν = 3304, 1741, 1624, 1595, 1524, 1481, 1408, 1255, 1152, 1086, 1028, 750, 698, 605 cm^{-1}.

Methyl N^2-((*S*)-1-((*R*)-2-((*R,S*)-2-hydroxy-3-(4-hydroxyphenyl)propanamido)-4-phenylbutanoyl)indoline-2-carbonyl)-N^ω-nitro-*L*-argininate (**10k**)

Synthesized from **9f** (100 mg, 0.186 mmol) using the general coupling procedure type C. White solid, yield: 65 mg (50%). ^{1}H NMR (DMSO-d_6, 400 MHz): δ 1.46-1.57 (m, 2H, $-CHCH_2\underline{CH_2}CH_2NH-$), 1.69-1.82 (m, 2H, $-CH\underline{CH_2}CH_2CH_2NH-$), 1.91-2.16 (m, 2H, $-CH(OH)\underline{CH_2}Ph(OH)$), 2.46-2.57 (m, 1H, $-CH\underline{CH_2}CH_2Ph$), 2.67-2.72 (m, 1H, $-CH\underline{CH_2}CH_2Ph$), 2.84-2.93 (m, 1H, $-CHCH_2\underline{CH_2}Ph$), 3.06-3.18 (m, 3H, $-CHCH_2CH_2\underline{CH_2}NH-$ + $-CHCH_2\underline{CH_2}Ph$), 3.55-3.72 (m, 4H, $-COO\underline{Me}$ + $-NHCH\underline{CH_2}-$), 4.05-4.14 (m, 1H, $-NHCH\underline{CH_2}-$), 4.21-4.28 (m, 1H, + $-\underline{CH}CH_2CH_2CH_2NH-$), 4.44-4.51(4.93-4.50) (m, 1H, $-\underline{CH}CH_2CH_2Ph$), 5.04-5.12 (m, 1H, $-NH\underline{CH}CH_2-$), 5.50 (5.67) (d, 1/2H, *J* = 6.0 Hz, $-\underline{CH}(OH)CH_2Ph(OH)$), 5.62(6.87) (d, 1/2H, *J* = 5.6 Hz, $\underline{CH}(OH)CH_2Ph(OH)$), 6.63-6.67 (m, 2H, $-CH(OH)CH_2\underline{Ph}(OH)$), 7.01-7.33 (m, 10H, indoline Ar-$\underline{H}$ + $-CHCH_2CH_2\underline{Ph}$ + $-CH(OH)CH_2\underline{Ph}(OH)$), 7.79-7.81(7.93-7.95) (m, 1H, -CO$\underline{NH}$-), 8.08-8.12 (m, 1H, indoline Ar-$\underline{H}$), 8.45-8.48 (8.76-8.80) (m, 1H, -CONH-), 8.56 (bs, 1H, guanidine-$\underline{NH}$), 9.12(9.13) (s, 1H, $-CH(OH)CH_2Ph(\underline{OH})$) ppm. HRMS for $C_{35}H_{40}N_7O_9$: calculated 702.2888; found 702.2896. IR(ATR): ν = 3298, 2946, 1739, 1628, 1594, 1514, 1482, 1410, 1347, 1258, 1105, 1080, 1023, 753, 700 cm^{-1}.

(*S*)-1-((*R*)-2-((*R,S*)-2-Hydroxy-3-(4-hydroxyphenyl)propanamido)-4-phenylbutanoyl)-*N*-((*S*)-1-hydroxy-5-(3-nitroguanidino)pentan-2-yl)indoline-2-carboxamide (**10l**)

Synthesized from **9h** (51mg, 0.10 mmol) using the general coupling procedure type C. White solid, yield: 36 mg (54%). ^{1}H NMR (DMSO-d_6, 400 MHz): δ 1.37-1.47 (m, 2H, $-CHCH_2\underline{CH_2}CH_2NH-$), 1.51-1.70 (m, 2H, $-CH\underline{CH_2}CH_2CH_2NH-$), 1.96-2.12 (m, 2H, $-CH(OH)\underline{CH_2}Ph(OH)$), 2.55-2.57 (m, 1H, $-CH\underline{CH_2}CH_2Ph$), 2.66-2.72 (m, 1H, $-CH\underline{CH_2}CH_2Ph$), 2.86-2.93 (m, 1H, $-CHCH_2\underline{CH_2}Ph$), 3.00-3.07 (m, 1H, $-CHCH_2\underline{CH_2}Ph$), 3.11-3.17 (m, 2H, $-CHCH_2CH_2\underline{CH_2}NH-$), 3.48-3.53 (m, 1H, $-\underline{CH_2}OH$), 3.62-3.71 (m,

2H -CH$_2$OH + -NHCHCH$_2$-), 4.06-4.12 (m, 1H, -NHCHCH$_2$-), 4.51-4.60 (m, 1H, -CHCH$_2$CH$_2$CH$_2$NH-), 4.66-4.72 (m, 1H, -CHCH$_2$CH$_2$Ph), 5.02-5.07 (m, 1H, -NHCHCH$_2$-), 5.64 (5.59) (d, 1H, *J* = 6.1 Hz, -CH(OH)CH$_2$Ph(OH)), 6.64-6.67 (m, 2H, -CH(OH)CH$_2$Ph(OH), 7.02-7.33 (m, 10H, indoline Ar-H + -CHCH$_2$CH$_2$Ph + -CH(OH)CH$_2$Ph(OH)), 7.78-7.82 (7.92-7.94) (m, 1H, -CONH-), 8.09-8.12 (m, 1H, indoline Ar-H), 8.18-8.23 (m, 1H, -CONH-), 8.55 (bs, 1H, guanidine-NH), 9.12(9.14) (s, 1H, -CH(OH)CH$_2$Ph(OH)) ppm. HRMS for $C_{34}H_{40}N_7O_8$: calculated 674.2938; found 674.2945. IR(ATR): *ν* = 3298, 2945, 1630, 1516, 1483, 1413, 1259, 1106, 1080, 1027, 828, 754, 700 cm^{-1}.

Methyl ((*R*)-1-((*R*)-2-((*R*)-2-hydroxy-3-phenylpropanamido)-4-phenylbutanoyl)indoline-2-carbonyl)-*L*-argininate (**11b**)

Synthesized from **10b** (50 mg, 0.485 mmol) using the general procedure of nitro group cleavage described in **6.10**. White solid, yield: 39 mg (78%). ^{1}H NMR (DMSO-*d*$_6$, 400 MHz): δ 1.56-1.62 (m, 2H, -CHCH$_2$CH$_2$CH$_2$NH-), 1.70-1.80 (m, 2H, -CHCH$_2$CH$_2$CH$_2$NH-), 1.83-1.95 (m, 2H, -CH(OH)CH$_2$Ph), 2.28-2.35 (m, 1H, -CHCH$_2$CH$_2$Ph), 2.47-2.57 (m, 1H, -CHCH$_2$CH$_2$Ph), 2.78 (dd, 1H, *J* = 13.8, *J* = 7.8 Hz, -CHCH$_2$CH$_2$Ph), 2.98 (dd, 1H, *J* = 13.7 Hz, *J* = 4.0 Hz, -CHCH$_2$CH$_2$Ph), 3.06-3.12 (m, 3H, -CHCH$_2$CH$_2$CH$_2$NH- + -NHCHCH$_2$-), 3.49-3.66 (m, 6H, -CH$_2$OH + -COOMe+ -NHCHCH$_2$-), 4.01-4.06 (m, 1H, -CHCH$_2$CH$_2$CH$_2$NH-), 4.18 (dd, 1H, *J* = 7.8 Hz, *J* = 4.1 Hz, -CHCH$_2$CH$_2$Ph), 4.27-4.33 (m, 1H, -NHCHCH$_2$-), 5.57 (dd, 1H, *J* = 11.1 Hz, *J* = 2.9 Hz, -CH(OH)CH$_2$Ph), 7.03 (dt, 1H, *J* = 7.5 Hz, *J* = 0.8 Hz, indoline Ar-H), 7.12-7.30 (m, 12H, indoline Ar-H + -CHCH$_2$CH2Ph + -CH(OH)CH$_2$Ph), 8.09 (d, 1H, *J* = 8.0 Hz, indoline Ar-H), 8.15 (d, 1H, *J* = 7.8 Hz, -CONH-), 8.47 (s, 1H, HCOOH), 9.18 (d, 1H, *J* = 6.5 Hz, -CONH-), 9.21 (t, 1H, *J* = 4.4 Hz, guanidine-NH) ppm. ^{13}C NMR (DMSO-*d*$_6$, 100 MHz): δ 173.49, 172.07, 171.57, 170.48, 167.06, 157.62, 143.06, 141.28, 138.17, 130.03, 129.62, 129.47, 128.24, 128.11, 127.82, 127.05, 125.96, 125.78, 124.55, 123.70, 116.47, 79.17, 71.84, 60.04, 52.42, 51.87, 50.90, 40.23, 33.97, 32.46, 31.54, 27.54, 24.89 ppm. HRMS for $C_{36}H_{43}N_6O_8$: calculated 687.3142; found 687.3158. IR(ATR): *ν* = 3197, 3060, 2952, 2780, 1742, 1638, 1595, 1481, 1454, 1349, 1271, 1211, 1090, 1024, 1003, 754, 699 cm^{-1}. HPLC: retention time 12.327 min, purity 99.0 % (254 nm).

(*R*)-*N*-((*S*)-5-Guanidino-1-hydroxypentan-2-yl)-1-((*R*)-2-((*R*)-2-hydroxy-3-phenylpropanamido)-3-phenylpropanoyl)indoline-2-carboxamide (**11c**)

Synthesized from **10c** (97 mg, 0.15 mmol) using the general procedure described in **6.10**. White solid, yield: 77 mg (79%). ^{1}H NMR (DMSO-*d*$_6$, 600 MHz): δ 1.38-1.40 (m, 1H, -CHCH$_2$CH$_2$CH$_2$NH-), 1.47-1.57 (m, 2H, -CHCH$_2$CH$_2$CH$_2$NH-), 1.63-1.68 (m, 1H, -CHCH$_2$CH$_2$CH$_2$NH-), 2.52-2.54 (m, 1H, -CH(OH)CH$_2$Ph), 2.76 (dd, 1H, *J* = 13.7 Hz, *J* = 3.9 Hz, -CH(OH)CH$_2$Ph), 2.97-3.00 (m, 4H, -CHCH$_2$Ph + -CHCH$_2$CH$_2$CH$_2$NH-), 3.14 (dd, 1H, *J* = 16.9 Hz, *J* = 1.9 Hz, -NHCHCH$_2$-), 3.30-3.33 (m, 1H, -CH$_2$OH), 3.40-3.42 (m, 1H, -CH$_2$OH), 3.59 (dd, 1H, *J* = 16.9 Hz, *J* = 11.3 Hz, -NHCHCH$_2$-), 3.67-3.72 (m, 1H, -CHCH$_2$CH$_2$CH$_2$NH-), 4.00 (dd, 1H, *J* = 8.3, *J* = 4.0 Hz, -CHCH$_2$Ph), 4.48 (dd, 1H, *J* = 13.8 Hz, *J* = 8.0 Hz, -NHCHCH$_2$-), 5.55 (dd, 1H, *J* = 11.2 Hz, *J* = 2.8 Hz, -CH(OH)CH$_2$Ph), 7.02-7.28 (m, 13H, indoline Ar-H + -CH(OH)CH$_2$Ph + -CHCH$_2$Ph), 8.14 (d, 1H, *J* = 8.2 Hz, -CONH-), 8.16 (d, 1H, *J* = 8.2 Hz, indoline Ar-H), 8.43 (s, 1H, $HCOO^-$), 8.52 (d, 1H, *J* = 7.8 Hz, -CONH-), 9.08 (t, 1H, *J* = 4.8 Hz, guanidine-NH) ppm. ^{13}C NMR (DMSO-d6, 150 MHz): δ 173.33, 171.10,170.47, 168.86, 157.59, 142.93, 138.08, 137.67, 130.15, 129.17, 128.97, 127.94, 127.68, 127.66, 126.93, 126.22, 125.78, 124.45, 123.58, 116.44, 71.83, 62.38, 60.51, 52.09, 51.26, 40.59, 40.228, 39.94, 36.21, 34.59, 27.57, 24.80 ppm. HRMS for $C_{33}H_{41}N_6O_5$: calculated 601.3138; found 601.3131. IR(ATR): *ν* = 3269, 3172, 2930, 2778, 1616, 1481, 1463, 1365, 1353, 1262, 1203, 1085, 773, 749, 698, 640 cm^{-1}. HPLC: retention time 11.889, purity 96.8 % (254 nm).

(*R*)-*N*-((*S*)-5-Guanidino-1-hydroxypentan-2-yl)-1-((*R*)-2-((*R*)-2-hydroxy-3-phenylpropanamido)-4-phenylbutanoyl)indoline-2-carboxamide (**11d**)

Synthesized from **10d** (40 mg, 0.06 mmol) using the general procedure of nitro group cleavage described in **6.10**. White solid, yield: 34 mg (85%). ^{1}H NMR (DMSO-*d*$_6$, 400 MHz): δ 1.39-1.67 (m, 4H, -CHCH$_2$CH$_2$CH$_2$NH- + -CHCH$_2$CH$_2$CH$_2$NH-), 1.87-1.95 (m, 2H, -CH(OH)CH$_2$Ph), 2.28-2.36 (m, 2H, -CHCH$_2$CH$_2$Ph), 2.78 (dd, 1H, *J* = 13.7 Hz, *J* = 8.0 Hz, -CHCH$_2$CH$_2$Ph), 2.95-3.11 (m, 4H, -CHCH$_2$CH$_2$Ph + -CHCH$_2$CH$_2$CH$_2$NH- + -CH$_2$OH), 3.29-3.42 (m, 2H, -CH$_2$OH +-NHCHCH$_2$-), 3.55-3.65 (m, 2H, -NHCHCH$_2$- + -CHCH$_2$CH$_2$CH$_2$NH-), 4.17 (dd, J = 7.7 Hz, J = 4.0 Hz, -CHCH$_2$CH$_2$Ph), 4.34-4.39 (d,

1H, $\mathrm{-NH\underline{CH}CH_2-}$), 5.48 (dd, 1H, *J* = 11.1 Hz, *J* = 2.5 Hz, $\mathrm{-\underline{CH}(OH)CH_2Ph}$), 7.02 (t, 1H, *J* = 7.3 Hz, indoline Ar-$\underline{\mathrm{H}}$), 7.10-7.31 (m, 12H, indoline Ar-$\underline{\mathrm{H}}$ + $\mathrm{-CHCH_2CH_2\underline{Ph}}$ + $\mathrm{-CH(OH)CH_2\underline{Ph}}$), 8.10 (d, 1H, *J* = 8.0 Hz, indoline Ar-$\underline{\mathrm{H}}$), 8.16 (d, 1H, *J* = 7.9 Hz, $\mathrm{-CO\underline{NH}-}$), 8.42-8.47 (m, 2H, $\mathrm{\underline{H}COOH}$ + $\mathrm{-CO\underline{NH}-}$), 9.03 (t, 1H, *J* = 4.7 Hz, guanidine-$\underline{\mathrm{NH}}$) ppm. ^{13}C NMR (MeOH-d_4, 100 MHz): δ 173.46, 171.07, 170.44, 166.37, 157.60, 143.13, 141.44, 138.16, 130.26, 129.59, 129.47, 128.28, 128.02, 127.81, 126.98, 125.95, 125.75, 124.51, 123.63, 116.54, 71.84, 62.55, 60.48, 51.18, 51.13, 40.64, 40.23, 34.63, 32.73, 31.64, 27.67, 24.90 ppm. HRMS for $C_{35}H_{43}N_6O_7$: calculated 659.3193; found 659.3178. IR(ATR): ν = 3297, 3028, 2929, 1623, 1535, 1482, 1408, 1364, 1259, 1085, 1027, 748, 698, 662, 604 cm^{-1}. HPLC: retention time 11.770 min, purity 95.4 % (254 nm).

Methyl ((*R*)-1-((*R*)-2-((*R,S*)-2-hydroxy-3-(4-hydroxyphenyl)propanamido)-4-phenylbutanoyl)indoline-2-carbonyl)-*L*-argininate (**11e**)

Synthesized from **10e** (50 mg, 0.071 mmol) using the general procedure of nitro group cleavage described in **6.10**. White solid, yield: 39 mg (78%). ^{1}H NMR (DMSO-d_6, 400 MHz): δ 1.54-1.64 (m, 2H, $\mathrm{-CHCH_2\underline{CH_2}CH_2NH-}$), 1.67-1.79 (m, 2H, $\mathrm{-CH\underline{CH_2}CH_2CH_2NH-}$), 1.86-1.95 (m, 2H, $\mathrm{-CH(OH)\underline{CH_2}Ph(OH)}$), 2.29-2.36 (m, 1H, $\mathrm{-CH\underline{CH_2}CH_2Ph}$), 2.55-2.70 (m, 1H, $\mathrm{-CH\underline{CH_2}CH_2Ph}$), 2.82-2.89 (m, 1H, $\mathrm{-CHCH_2\underline{CH_2}Ph}$), 3.10-3.16 (m, 3H, $\mathrm{-CHCH_2CH_2\underline{CH_2}NH-}$ + $\mathrm{-CHCH_2\underline{CH_2}Ph}$), 3.59-3.64 (m, 4H, $\mathrm{-COO\underline{Me}}$ + $\mathrm{-NHCH\underline{CH_2}-}$), 4.02-4.12 (m, 2H, $\mathrm{-NHCH\underline{CH_2}-}$ + $\mathrm{-\underline{CH}CH_2CH_2CH_2NH-}$), 4.22-4.32 (m, 1H, $\mathrm{-\underline{CH}CH_2CH_2Ph}$), 4.99-5.10 (m, 1H, $\mathrm{-NH\underline{CH}CH_2-}$), 5.55-5.61 (m, 1H, $\mathrm{-\underline{CH}(OH)CH_2Ph(OH)}$), 6.59-6.62 (m, 2H, $\mathrm{-CH(OH)CH_2\underline{Ph}(OH)}$), 6.98-7.30 (m, 10H, indoline Ar-$\underline{\mathrm{H}}$ + $\mathrm{-CHCH_2CH_2\underline{Ph}}$ + $\mathrm{-CH(OH)CH_2\underline{Ph}(OH)}$), 8.08-8.11(8.27-8.29) (m, 2H, $\mathrm{-CO\underline{NH}-}$ + -indoline Ar-$\underline{\mathrm{H}}$), 8.38 (s, 1H, $\mathrm{\underline{H}COOH}$), 8.96-8.98 (m, 1H, $\mathrm{-CO\underline{NH}-}$), 9.10(9.12) (s, 1H, $\mathrm{-CH(OH)CH_2Ph(\underline{OH})}$) ppm. ^{13}C NMR (DMSO-$d_6$, 100 MHz): δ 173.86, 173.69, 172.07, 171.59, 171.56, 170.57, 170.52, 165,73, 157.46, 155.77, 155.72, 143.08, 143.04, 141.26, 130.32, 130.16, 130.01, 129.98, 128.42, 128.37, 128.24, 128.10, 128.00, 127.07, 125.77, 124.56, 123.69, 116.46, 114.72, 114.68, 72.27, 72.11, 60.03, 52.39, 51.89, 51.03, 50.90, 48.57, 40.15, 33.97, 32.43, 31.56, 27.54, 24.96 ppm. HRMS for $C_{36}H_{43}N_6O_9$: calculated 703.3092; found 703.3076. IR(ATR): ν = 3203, 2953, 2816, 2778, 1739, 1630, 1514, 1482, 1353, 1215, 1105, 752, 699 cm^{-1}. HPLC: retention time 11.417 min and 11.453, purity 99.1 % (254 nm).

(*R*)-*N*-((*S*)-5-Guanidino-1-hydroxypentan-2-yl)-1-((*R*)-2-((*R,S*)-2-hydroxy-3-(4-hydroxyphenyl)propanamido)-4-phenylbutanoyl)indoline-2-carboxamide (**11f**)

Synthesized from **10f** (26 mg, 0.04 mmol) using the general procedure of nitro group cleavage described in **6.10**. White solid, yield: 22 mg (81%). ^{1}H NMR (DMSO-d_6, 400 MHz): δ 1.39-1.51 (m, 2H, $\mathrm{-CHCH_2\underline{CH_2}CH_2NH-}$), 1.60-1.67 (m, 2H, $\mathrm{-CH\underline{CH_2}CH_2CH_2NH-}$), 1.80-1.99 (m, 2H, $\mathrm{-CH(OH)\underline{CH_2}Ph(OH)}$), 2.30-2.37 (m, 1H, $\mathrm{-CH\underline{CH_2}CH_2Ph}$), 2.46-2.57 (m, 1H, $\mathrm{-CH\underline{CH_2}CH_2Ph}$), 2.60-2.70 (m, 1H, $\mathrm{-CHCH_2\underline{CH_2}Ph}$), 2.81-2.88 (m, 1H, $\mathrm{-CHCH_2\underline{CH_2}Ph}$), 3.04-3.11 (m, 3H, $\mathrm{-CHCH_2CH_2\underline{CH_2}NH-}$ + $\mathrm{-NHCH\underline{CH_2}-}$), 3.31 (dd, 1H, *J* = 10.9 Hz, *J* = 5.7 Hz, $\mathrm{-\underline{CH_2}OH}$), 3.40 (dd, 1H, *J* = 10.8 Hz, *J* = 4.8 Hz, $\mathrm{-\underline{CH_2}OH}$), 3.56-3.66 (m, 2H, $\mathrm{-NHCH\underline{CH_2}-}$ + $\mathrm{-\underline{CH}CH_2CH_2CH_2NH-}$), 4.07-4.11 (m, 1H, $\mathrm{-\underline{CH}CH_2CH_2Ph}$), 4.29-4.38 (m, 1H, $\mathrm{-NH\underline{CH}CH_2-}$), 5.46-5.52 (m, 1H, $\mathrm{-\underline{CH}(OH)CH_2Ph(OH)}$), 6.59-6.62 (m, 2H, $\mathrm{-CH(OH)CH_2\underline{Ph}(OH)}$, 6.97-7.30 (m, 10H, indoline Ar-$\underline{\mathrm{H}}$ + $\mathrm{-CHCH_2CH_2\underline{Ph}}$ + $\mathrm{-CH(OH)CH_2\underline{Ph}(OH)}$), 8.09-8.12 (m, 3/2H, indoline Ar-$\underline{\mathrm{H}}$ + $\mathrm{-CO\underline{NH}-}$), 8.25-8.27 (m, 1/2H, -CONH-), 8.41-8.42 (m, 2H, $\mathrm{\underline{H}COOH}$ + $\mathrm{-CO\underline{NH}-}$), 8.90(8.91) (s, 1H, $\mathrm{-CH(OH)CH_2Ph(\underline{OH})}$) ppm. ^{13}C NMR (DMSO-$d_6$, 100 MHz): δ 173.86, 173.56, 171.12, 171.08, 170.49, 166.20, 157.52, 155.81, 155.76, 143.15, 143.11, 141.45, 130.30, 130.25, 130.22, 130.16, 128.38, 128.28, 128.17, 128.01, 127.00, 125.73, 124.51, 123.61, 116.52, 114.71, 72.30, 72.13, 62.60, 60.46, 51.33, 51.19, 51.10, 48.57, 40.63, 34.63, 32.72, 31.69, 31.65, 27.67, 24.97 ppm. HRMS for $C_{34}H_{43}N_6O_6$: calculated 631.3244; found 631.3249. IR(ATR): ν = 3276, 3204, 2924, 2815, 2778, 1623, 1514, 1482, 1367, 1353, 1247, 1105, 751, 699, 656 cm^{-1}. HPLC: retention time 11.830 min and 10.900 min, purity 99.2 % (254 nm).

Methyl ((*S*)-1-(((*R*)-2-hydroxy-3-phenylpropanoyl)-*D*-phenylalanyl)indoline-2-carbonyl)-*L*-argininate (**11g**)

Synthesized from **10g** (135 mg, 0.2 mmol) using the general procedure of nitro group cleavage described in **6.10**. White solid, yield: 108 mg (80%). $[\alpha]^{25}_{D}$ +0.73 (c 0.137, MeOH), ^{1}H NMR (DMSO-d_6, 600 MHz): δ 1.48-1.53 (m, 2H, $\mathrm{-CHCH_2\underline{CH_2}CH_2NH-}$), 1.67-1.72 (m, 1H, $\mathrm{-CH\underline{CH_2}CH_2CH_2NH-}$), 1.73-1.81 (m, 1H, $\mathrm{-CH\underline{CH_2}CH_2CH_2NH-}$), 2.66 (dd, 1H, *J* = 13.8 Hz, *J* = 8.4 Hz, $\mathrm{-CH(OH)\underline{CH_2}Ph}$), 2.92 (dd, 1H, *J* = 13.8 Hz, *J* = 3.1 Hz, $\mathrm{-CH(OH)\underline{CH_2}Ph}$), 2.96 (dd, 1H, *J* = 5.8 Hz, *J* = 2.2 Hz, $\mathrm{-CH\underline{CH_2}Ph}$), 3.02-3.06 (m, 3H, -

CH$\underline{\text{CH}_2}$Ph + -CHCH$_2$CH$_2$$\underline{\text{CH}_2}$NH-), 3.39 (dd, 1H, *J* = 16.5 Hz, *J* = 10.8 Hz, -NHCH$\underline{\text{CH}_2}$-), 3.59 (s, 3H, -COO$\underline{\text{Me}}$), 3.59-3.71 (m, 1H, -NHCH$\underline{\text{CH}_2}$-), 4.07 (dd, 1H, *J* = 8.3 Hz, *J* = 3.3 Hz, -$\underline{\text{CH}}$CH$_2$CH$_2$CH$_2$NH-), 4.14-4.18 (m, 1H, -$\underline{\text{CH}}$CH$_2$Ph), 4.62 (dd, 1H, *J* = 13.7 Hz, *J* = 6.5 Hz, -NH$\underline{\text{CH}}$CH$_2$-), 4.87 (dd, 1H, *J* = 10.6 Hz, *J* = 2.2 Hz, -$\underline{\text{CH}}$(OH)CH$_2$Ph), 7.00-7.03 (m, 3H, indoline Ar-$\underline{\text{H}}$ + -CHCH$_2$$\underline{\text{Ph}}$), 7.16-7.28 (m, 10H, indoline Ar-$\underline{\text{H}}$ + -CH(OH)CH$_2$$\underline{\text{Ph}}$ + -CHCH$_2$$\underline{\text{Ph}}$), 7.68 (d, 1H, *J* = 7.6 Hz, -CO$\underline{\text{NH}}$-), 8.03 (d, 1H, *J* = 8.1 Hz, indoline Ar-$\underline{\text{H}}$), 8.44 (s, 1H, $\underline{\text{H}}$COO$^-$), 9.09 (d, 1H, *J* = 7.2 Hz, -CO$\underline{\text{NH}}$-) ppm. ^{13}C NMR (DMSO-*d*$_6$, 150 MHz): δ 171.92, 171.69, 170.43, 169.37, 166.89, 157.49, 142.60, 138.30, 135.85, 129.81, 129.48, 129.41, 127.94, 127.80, 127.05, 126.50, 125.92, 124.42, 123.78, 116.27, 71.76, 60.16, 52.18, 51.83, 40.16, 40.00, 39.94, 38.37, 34.15, 27.82, 24.83 ppm. HRMS for $C_{34}H_{41}N_6O_6$: calculated 629.3088; found 629.3083. IR(ATR): ν = 3350, 2954, 1741, 1649, 1523, 1481, 1415, 1346, 1200, 1178, 1131, 1087, 1029, 835, 800, 753, 720, 699 cm^{-1}. HPLC: retention time 12.350 min, purity 97.2 % (254 nm).

Methyl ((*S*)-1-((*R*)-2-((*R*)-2-hydroxy-3-phenylpropanamido)-4-phenylbutanoyl)indoline-2-carbonyl)-*L*-argininate (**11h**)

Synthesized from **10h** (60 mg, 0.087 mmol) using the general procedure of nitro group cleavage described in **6.10**. White solid, yield: 50 mg (83%). ^{1}H NMR (DMSO-*d*$_6$, 400 MHz): δ 1.48-1.53 (m, 3H, -CHCH$_2$$\underline{\text{CH}_2}CH_2$NH- + -CH$\underline{\text{CH}_2}CH_2CH_2$NH-), 1.66-1.85 (m, 3H, -CH$\underline{\text{CH}_2}$CH$_2$CH$_2$NH- + -CH(OH)$\underline{\text{CH}_2}$Ph), 2.01-2.12 (m, 1H, -CH$\underline{\text{CH}_2}$CH$_2$Ph), 2.45-2.49 (m, 1H, -CH$\underline{\text{CH}_2}$CH$_2$Ph), 2.80 (dd, 1H, *J* = 13.8 Hz, *J* = 7.9 Hz, -CHCH$_2$$\underline{\text{CH}_2}$Ph), 3.00-3.09 (m, 4H, -CHCH$_2$$\underline{\text{CH}_2}$Ph + -CHCH$_2CH_2$$\underline{\text{CH}_2}$NH- + -NHCH$\underline{\text{CH}_2}$-), 3.40-3.47 (m, 1H, -$\underline{\text{CH}_2}$OH), 3.60-3.69 (m, 4H, -$\underline{\text{CH}_2}$OH+ -COO$\underline{\text{Me}}$), 4.15-4.24 (m, 3H, -NHCH$\underline{\text{CH}_2}$- + -$\underline{\text{CH}}$CH$_2$CH$_2$CH$_2$NH- + -$\underline{\text{CH}}$CH$_2$CH$_2$Ph), 4.46-4.50 (m, 1H, -NH$\underline{\text{CH}}$CH$_2$-), 5.12 (dd, 1H, *J* = 10.5 Hz, *J* = 2.3 Hz, -$\underline{\text{CH}}$(OH)CH$_2$Ph), 7.04 (t, 1H, *J* = 7.4 Hz, indoline Ar-$\underline{\text{H}}$), 7.15-7.31 (m, 12H, indoline Ar-$\underline{\text{H}}$ + -CHCH$_2$CH2$\underline{\text{Ph}}$ + -CH(OH)CH$_2$$\underline{\text{Ph}}$), 7.85 (d, 1H, *J* = 7.6 Hz, -CO$\underline{\text{NH}}$-), 8.10 (d, 1H, *J* = 8.1 Hz, indoline Ar-$\underline{\text{H}}$), 8.42 (s, 1H, $\underline{\text{H}}$COOH), 9.04-9.08 (m, 2H, -CONH- + guanidine-$\underline{\text{NH}}$) ppm. HRMS for $C_{36}H_{43}N_6O_8$: calculated 687.3142; found 687.3158. IR(ATR): ν = 3198, 3061, 2951, 1741, 1641, 1594, 1481, 1453, 1409, 1346, 1266, 1212, 1090, 1023, 1004, 822, 754, 699 cm^{-1}. HPLC: retention time 12.608 min, purity 99.5 % (254 nm).

(*S*)-*N*-((*S*)-5-Guanidino-1-hydroxypentan-2-yl)-1-((*R*)-2-((*R*)-2-hydroxy-3-phenylpropanamido)-3-phenylpropanoyl)indoline-2-carboxamide (**11i**)

Synthesized from **10i** (51 mg, 0.14 mmol) using the general procedure described in **6.10**. White solid, yield: 72 mg (80%). ^{1}H NMR (DMSO-*d*$_6$, 600 MHz): δ 1.40-1.42 (m, 2H, -CHCH$_2$$\underline{\text{CH}_2}CH_2$NH-), 1.50-1.53 (m, 1H, -CH$\underline{\text{CH}_2}$CH$_2$CH$_2$NH-), 1.64-1.68 (m, 1H, -CH$\underline{\text{CH}_2}$CH$_2$CH$_2$NH-), 2.62 (dd, 1H, *J* = 13.8 Hz, *J* = 8.4 Hz, -CH(OH)$\underline{\text{CH}_2}$Ph), 2.85-2.92 (m, 3H, -CH(OH)$\underline{\text{CH}_2}$Ph + -CH$\underline{\text{CH}_2}$Ph), 3.00-3.06 (m, 3H, -CHCH$_2$CH$_2$$\underline{\text{CH}_2}$NH- + -NHCH$\underline{\text{CH}_2}$-), 3.31 (dd, 1H, *J* = 10.8 Hz, *J* = 6.7 Hz, -$\underline{\text{CH}_2}$OH), 3.41 (dd, 1H, *J* = 16.6 Hz, *J* = 10.8 Hz, -$\underline{\text{CH}_2}$OH), 3.48 (dd, 1H, *J* = 10.8 Hz, *J* = 4.0 Hz, -NHCH$\underline{\text{CH}_2}$-), 3.57-3.62 (m, 1H, -$\underline{\text{CH}}$CH$_2$CH$_2$CH$_2$NH-), 4.05 (dd, 1H, *J* = 8.3 Hz, *J* = 3.3 Hz, -$\underline{\text{CH}}$CH$_2$Ph), 4.70 (dd, 1H, *J* = 14.6 Hz, *J* = 6.6 Hz, -NH$\underline{\text{CH}}$CH$_2$-), 4.90 (dd, 1H, *J* = 10.7 Hz, *J* = 2.6 Hz, -$\underline{\text{CH}}$(OH)CH$_2$Ph), 7.00-7.04 (m, 3H, indoline Ar-$\underline{\text{H}}$ + -CHCH$_2$$\underline{\text{Ph}}$), 7.15-7.29 (m, 10H, indoline Ar-$\underline{\text{H}}$ + -CH(OH)CH$_2$$\underline{\text{Ph}}$ + -CHCH$_2$$\underline{\text{Ph}}$), 7.65 (d, 1H, *J* = 8.3 Hz, -CO$\underline{\text{NH}}$-), 8.04 (d, 1H, *J* = 8.1 Hz, indoline Ar-$\underline{\text{H}}$), 8.46 (s, 1H, $\underline{\text{H}}$COO$^-$), 8.55 (d, 1H, *J* = 8.1 Hz, -CO$\underline{\text{NH}}$-), 9.13 (t, 1H, *J* = 6.1 Hz, guanidine-$\underline{\text{NH}}$) ppm. ^{13}C NMR (DMSO-d6, 150 MHz): δ 171.97, 169.99, 169.32, 166.50, 157.53, 142.82, 138.27, 135.81, 130.03, 129.48, 129.45, 129.43, 127.86, 127.81, 127.79, 127.78, 126.99, 126.43, 125.94, 124.43, 123.67, 116.06, 71.76, 62.76, 60.20, 51.33, 50.70, 40.45, 40.16, 39.94, 34.32, 27.69, 24.87 ppm. HRMS for $C_{33}H_{41}N_6O_5$: calculated 601.3138; found 601.3131. IR(ATR): ν = 3269, 3063, 2925, 2778, 1623, 1481, 1454, 1366, 1353, 1266, 1215, 1087, 1024, 1002, 773, 755, 699 cm^{-1}. HPLC: retention time 11.371, purity 97.5 % (254 nm).

(*S*)-*N*-((*S*)-5-Guanidino-1-hydroxypentan-2-yl)-1-((*R*)-2-((*R*)-2-hydroxy-3-phenylpropanamido)-4-phenylbutanoyl)indoline-2-carboxamide (**11j**)

Synthesized from **10j** (33 mg, 0.05 mmol) using the general procedure of nitro group cleavage described in **6.10**. White solid, yield: 26 mg (80%). ^{1}H NMR (DMSO-*d*$_6$, 400 MHz): δ 1.48-1.54 (m, 2H, -CHCH$_2$$\underline{\text{CH}_2}CH_2$NH-), 1.64-1.68 (m, 1H, -CH$\underline{\text{CH}_2}$CH$_2$CH$_2$NH-), 1.74-1.81 (m, 1H, -CH$\underline{\text{CH}_2}$CH$_2$CH$_2$NH-), 1.94-2.06 (m, 2H, -CH(OH)$\underline{\text{CH}_2}$Ph), 2.40-2.48 (m, 2H, -CH$\underline{\text{CH}_2}$CH$_2$Ph), 2.81 (dd, 1H, *J* = 13.8 Hz, *J* = 7.8

Hz, -CHCH$_2$$\underline{CH_2}$Ph), 3.00-3.08 (m, 4H, -CHCH$_2$$\underline{CH_2}$Ph + -CHCH$_2CH_2$$\underline{CH_2}$NH- + -$\underline{CH_2}$OH), 3.35 (dd, 1H, *J* = 10.9 Hz, *J* = 6.6 Hz, -$\underline{CH_2}$OH), 3.52 (dd, 1H, *J* = 10.9 Hz, *J* = 3.8 Hz, -NHCH$\underline{CH_2}$-), 3.63-3.69 (m, 1H, -NHCH$\underline{CH_2}$-), 4.16-4.19 (m, 2H, -$\underline{CH}$CH$_2$CH$_2$CH$_2$NH- + -$\underline{CH}$CH$_2$CH$_2$Ph), 4.52-4.57 (d, 1H, -NH$\underline{CH}$CH$_2$-), 5.07 (dd, 1H, *J* = 11.0 Hz, *J* = 2.6 Hz, -$\underline{CH}$(OH)CH$_2$Ph), 7.03 (dt, 1H, *J* = 7.4 Hz, *J* = 0.8 Hz, indoline Ar-$\underline{H}$), 7.15-7.29 (m, 12H, indoline Ar-$\underline{H}$ + -CHCH2CH2$\underline{Ph}$ + -CH(OH)CH$_2$$\underline{Ph}$), 7.81 (d, 1H, *J* = 8.1 Hz, -CO$\underline{NH}$-), 8.10 (d, 1H, *J* = 8.1 Hz, indoline Ar-$\underline{H}$), 8.44 (s, 1H, $\underline{H}$COOH), 8.50 (d, 1H, J = 8.0 Hz, -CO$\underline{NH}$-), 9.09 (t, 1H, *J* = 4.5, *J* = 4.5 Hz, guanidine-$\underline{NH}$) ppm. HRMS for $C_{35}H_{43}N_6O_7$: calculated 659.3193; found 659.3178. IR(ATR): ν = 3338, 2929, 2778, 1614, 1481, 1453, 1410, 1365, 1315, 1260, 1087, 1028, 753, 728, 698 cm^{-1}. HPLC: retention time 12.157 min, purity 90.3 % (254 nm).

Methyl ((2*S*)-1-((2*R*)-2-((*R*,*S*)-2-hydroxy-3-(4-hydroxyphenyl)propanamido)-4-phenylbutanoyl)indoline-2-carbonyl)-*L*-argininate (**11k**)

Synthesized from **10k** (50 mg, 0.071 mmol) using the general procedure of nitro group cleavage described in **6.10**. White solid, yield: 41 mg (83%). ^{1}H NMR (DMSO-d_6, 400 MHz): δ 1.46-1.55 (m, 2H, -CHCH$_2$$\underline{CH_2}CH_2$NH-), 1.69-1.79 (m, 2H, -CH$\underline{CH_2}$CH$_2$CH$_2$NH-), 1.89-2.16 (m, 2H, -CH(OH)$\underline{CH_2}$Ph(OH)), 2.46-2.56 (m, 1H, -CH$\underline{CH_2}$CH$_2$Ph), 2.65-2.71 (m, 1H, -CH$\underline{CH_2}$CH$_2$Ph), 2.85-2.93 (m, 1H, -CHCH$_2$$\underline{CH_2}$Ph), 3.00-3.14 (m, 3H, -CHCH$_2$CH$_2$$\underline{CH_2}$NH- + -CHCH$_2$$\underline{CH_2}$Ph), 3.61-3.68 (m, 4H, -COO$\underline{Me}$ + -NHCH$\underline{CH_2}$-), 4.06-4.12 (m, 3/2H, -NHCH$\underline{CH_2}$- + -$\underline{CH}$CH$_2$CH$_2$CH$_2$NH-), 4.06-4.12 (m, 3/2H, -$\underline{CH}$CH$_2$CH$_2$CH$_2$NH- + -$\underline{CH}$CH$_2$CH$_2$Ph), 4.45-4.49 (m, 1H, -NH$\underline{CH}$CH$_2$-), 5.07-5.12 (m, 1H, -$\underline{CH}$(OH)CH$_2$Ph(OH)), 6.64-6.67 (m, 2H, -CH(OH)CH$_2$$\underline{Ph}$(OH)), 7.02-7.33 (m, 10H, indoline Ar-$\underline{H}$ + -CHCH$_2$CH$_2$$\underline{Ph}$ + -CH(OH)CH$_2$$\underline{Ph}$(OH)), 7.82-7.83(7.95-8.02) (m, 1H, -indoline Ar-$\underline{H}$), 8.08-8.11(8.13-8.16) (m, 1H, -CO$\underline{NH}$-), 8.40 (s, 1H, $\underline{H}$COOH), 8.91-9.00 (m, 2H, -CO$\underline{NH}$- + -CH(OH)CH$_2$Ph($\underline{OH}$)) ppm. HRMS for $C_{36}H_{43}N_6O_9$: calculated 703.3092; found 703.3076. IR(ATR): ν = 3204, 2951, 2825, 1737, 1648, 1594, 1514, 1482, 1412, 1386, 1348, 1215, 1105, 754, 700 cm^{-1}. HPLC: retention time 11.606 min and 11.643 min, purity 99.6 % (254 nm).

(2*S*)-*N*-((*S*)-5-guanidino-1-hydroxypentan-2-yl)-1-((2*R*,*S*)-2-(2-hydroxy-3-(4-hydroxyphenyl)propanamido)-4-phenylbutanoyl)indoline-2-carboxamide *(***11l***)*

Synthesized from **10l** (26 mg, 0.04 mmol) using the general procedure of nitro group cleavage described in **6.10**. White solid, yield: 23 mg (84%). ^{1}H NMR (DMSO-d_6, 400 MHz): δ 1.37-1.67 (m, 4H, -CH$\underline{CH_2CH_2}$CH$_2$NH-), 1.76-1.91 (m, 1H, CH(OH)$\underline{CH_2}$Ph(OH)), 1.96-2.12 (m, 1H, -CH(OH)$\underline{CH_2}$Ph(OH)), 2.47-2.62 (m, 2H, -CH$\underline{CH_2}$CH$_2$Ph), 2.85-2.94 (m, 2H, -CHCH$_2$$\underline{CH_2}$Ph), 3.00-3.13 (m, 3H, -CHCH$_2$CH$_2$$\underline{CH_2}$NH- + -NHCH$\underline{CH_2}$-), 3.33-3.42 (m, 1H, -$\underline{CH_2}$OH), 3.49-3.53 (m, 1H, -$\underline{CH_2}$OH), 3.63-3.70 (m, 2H, -NHCH$\underline{CH_2}$- + -$\underline{CH}$CH$_2$CH$_2$CH$_2$NH-), 4.07-4.13 (m, 1H, -$\underline{CH}$CH$_2$CH$_2$Ph), 4.52-4.59 (m, 1H, -NH$\underline{CH}$CH$_2$-), 5.04-5.09 (m, 1H, -$\underline{CH}$(OH)CH$_2$Ph(OH)), 6.65-6.68 (m, 2H, -CH(OH)CH$_2$$\underline{Ph}$(OH)), 7.03-7.33 (m, 10H, indoline Ar-$\underline{H}$ + -CHCH$_2$CH$_2$$\underline{Ph}$ + -CH(OH)CH$_2$$\underline{Ph}$(OH)), 7.81(7.95) (d, 1H, *J* = 8.2 Hz, -CO$\underline{NH}$-), 8.10-8.12 (m, 1H, indoline Ar-$\underline{H}$), 8.41-8.49 (m, 2H, $\underline{H}$COOH + -CO$\underline{NH}$-), 8.93(8.95) (s, 1H, -CH(OH)CH$_2$Ph($\underline{OH}$)) ppm. ^{13}C NMR (DMSO-d_6, 100 MHz): δ 173.22, 172.77, 170.43, 170.23, 170.18, 170.02, 166.03, 157.45, 155.79, 155.71, 143.12, 141.10, 140.94, 131.95, 130.42, 130.35, 130.20, 128.82, 128.50, 128.41, 128.26, 128.17, 127.06, 126.06, 125.85, 125.81, 125.72, 124.53, 123.80, 116.28, 116.18, 114.81, 114, 71, 113.36, 72.36, 72.19, 63.08, 62.78, 61.06, 60.16, 60.10, 50.79, 50.71, 50.44, 50.27, 48.57, 40.55, 34.74, 34.49, 34.41, 30.94, 30.90, 30.67, 27.83, 25.08, 25.04, 24.92 ppm. HRMS for $C_{34}H_{43}N_6O_6$: calculated 631.3244; found 631.3249. IR(ATR): ν = 3193, 2943, 2779, 1616, 1593, 1514, 1481, 1415, 1370, 1350, 1245, 1171, 1105, 1080, 753, 699, 618 cm^{-1}. HPLC: retention time 11.054 and 11.168, purity 98.4 % (254 nm).
